# A mixed methods pilot randomised controlled trial to develop and evaluate the feasibility of a Mediterranean diet and lifestyle education intervention ‘THINK-MED’ among people with cognitive impairment

**DOI:** 10.1186/s40814-020-00738-3

**Published:** 2021-01-04

**Authors:** Andrea M. McGrattan, Claire T. McEvoy, Aswathy Vijayakumar, Sarah E. Moore, Charlotte E. Neville, Bernadette McGuinness, Michelle C. McKinley, Jayne V. Woodside

**Affiliations:** Centre for Public Health, Queens University Belfast, Belfast, BT12 6BJ UK

**Keywords:** Mediterranean diet, Intervention, Cognitive impairment, Prevention, Feasibility

## Abstract

**Background:**

Accumulating evidence supports the role of a Mediterranean diet (MD) in cognition, with a greater adherence to a MD associated with reduced dementia risk. It is important to understand how best to achieve behaviour change towards a MD, particularly in non-Mediterranean and high-risk populations. The aim of this study was to evaluate the feasibility of a MD and lifestyle education intervention (THINK-MED) among older adults with cognitive impairment.

**Methods:**

Qualitative interviews (Phase I), conducted with mild cognitive impairment (MCI) participants, evaluated the intervention materials to allow refinement before pilot testing. THINK-MED was a 12-month, pilot RCT (Phase II) whereby MCI participants were randomised into one of three groups: education on one occasion vs education staged and supported by a dietitian vs control. The primary outcome was a change in MD score. Secondary outcome measurements were collected to gather data on variability of the outcomes to inform a full trial power calculation and to test their acceptability. Slower recruitment rates necessitated a change in primary outcome to a revised focus primarily on feasibility. A sub-study of the same THINK-MED intervention and data collection measures was also conducted among community-dwelling participants with subjective cognitive impairment (SCI) (NCT03569319).

**Results:**

A total of 20 participants (*n* = 15 MCI; *n* = 5 SCI) were enrolled in the THINK-MED study. However, there were 10 (50%) participants (*n* = 9 MCI; *n* = 1 SCI) who withdrew from the study. Although, those who remained in the intervention rated and evaluated their experience as generally positive, recruitment and retention, especially from a MCI population, was extremely challenging. This highlights the complex needs of this heterogeneous clinical cohort. Recruiting participants from a community-based sample with SCI was more feasible, reaching a wider audience in a shorter time period. There were challenges relating to data collection and incompleteness, with the occurrence of missing data particularly among the questionnaire-based assessments.

**Conclusions:**

Owing to the challenges with recruitment and retention of older adults with cognitive impairment in this study, it is difficult to confirm if this intervention is feasible. This research has highlighted considerations for a future trial, including a review of the recruitment strategies used and prioritisation of the outcome measures assessed.

**Trial registration:**

Clinical trials registration NCT03265522/NCT03569319

**Supplementary Information:**

The online version contains supplementary material available at 10.1186/s40814-020-00738-3.

## Key messages

What was known about feasibility prior to this study?

- Limited evidence on recruitment, retention and follow-up rates of MCI/SCI participants in dietary intervention studies and their ability to change dietary behaviours
- A scarcity of large-scale dietary intervention trials in MCI/SCI to identify an indicative sample size to determine efficacy

What are the findings on the feasibility from this study?

- Recruitment and retention from a MCI clinical cohort was challenging; recruitment from a community setting of people at risk of cognitive decline was more promising
- Uncertain whether a MD dietary intervention is feasible and acceptable among people with/at risk of cognitive impairment
- Participation, retention and engagement in this dietary intervention study was heterogeneous and seemed to depend on individual circumstances, e.g. the presence of co-morbidities and motivation to change behaviours

What are the implications for the design of the main study?

- Further pilot testing required before a definitive trial can be implemented
- Consideration of the sampling frame used (clinical vs community) for recruitment
- Review of the appropriateness of the inclusion and exclusion criteria for entry into the study
- Prioritisation of outcome measures required to reduce participant burden and minimise occurrence of missing data

## Introduction

Dementia was reported to affect 47 million people worldwide in 2017, with projections estimating this figure to nearly triple by 2050 [1]. With a lack of pharmacological treatment for impaired cognition, research attention has been centred on the role of modifiable risk factors in an attempt to improve cognitive health outcomes. Diet has been suggested to be significantly associated with reduced dementia risk, particularly owing to the influence of dietary components on the inflammatory mechanisms and oxidative stress involved in the pathophysiology of cognitive decline [2, 3], as well as processes such as neurogenesis and neuronal connectivity involved in the function of the brain [4].

Mild cognitive impairment (MCI) has been described as ‘a symptomatic pre-dementia stage’ characterised as an intermediate phase between the associated decline of normal aging and that of dementia [5]. Although these individuals are on a continuum between normal aging and pathological cognitive decline, MCI has been highlighted as a manageable, or even potentially reversible, state and thus could be a prime target for therapeutic prevention strategies in order to delay progression to dementia [6]. Healthy dietary patterns such as the traditional Mediterranean diet (MD), characterised by a high intake of fruits, vegetables, wholegrains, nuts, legumes and olive oil, a moderate intake of fish, poultry and alcohol and a low intake of red meat, have been linked with improved health outcomes, including benefits to cardiovascular health as well as improved cognition [7–11]. A greater adherence to the MD has been associated with better cognitive performance [12–17], a lower risk of cognitive impairment [12, 18–20] and Alzheimer’s disease (AD) [21] as well as reduced rate of cognitive decline [20, 22–24]. There have been inconsistencies in the literature, however, [25–29] possibly due to methodological differences between studies in terms of the populations studied and methods used to assess diet and cognition. It is possible that diet induces only subtle effects on cognition that are difficult to detect in cognitively healthy adults, especially over short time-frames; therefore, the sample population that is tested is of importance. There is promising accumulating evidence from observational studies that report a link between greater MD adherence and promoting brain structures and functions that protect against neurodegeneration including increased cortical thickness [30, 31], greater brain volumes [32], slower rate of hippocampal atrophy and improved structural connectivity [33–35] as well as less amyloid (Aβ) accumulation at both midlife and older age [36, 37]. It is therefore important to develop targeted and tailored interventions, particularly in non-Mediterranean countries and among those at risk of cognitive decline, to further explore the diet and cognition link as well as fully understand and identify the ability of individuals to incorporate this dietary pattern into their own lifestyles and make dietary behaviour changes.

It is vital that interventions are thoroughly planned, piloted and evaluated before implementation to ensure the most effective methods have been chosen for the specific target group. This is reinforced by the Medical Research Council (MRC) guidance on developing complex interventions, which outlines key steps in designing and developing effective health promotion interventions [38, 39]. The guidance suggests that by employing a theoretically driven, systematic approach to the design and planning of an intervention, researchers are helped to tailor the intervention to the target population as well as provide key information to interpret the effects of the study, to inform evaluation and, thereafter, the development of future research [38, 39]. We developed educational resources related to the Mediterranean diet and lifestyle (THINK-MED) in accordance with the MRC guidance for developing and evaluating complex interventions [38, 39], informed by a systematic literature review [40] and previous qualitative work with patients with MCI who evaluated a first version of MD educational material [41]. The aims of the multi-phase research study presented here were to conduct further qualitative work among a cohort of MCI participants (Phase I) in order to evaluate the acceptability of the refined THINK-MED resources and overall dietary intervention and to understand the barriers and enablers to dietary change among people with mild memory problems. The feedback gathered in this phase informed further refinement and tailoring of the resource and contributed to the overall design of the THINK-MED dietary intervention. In Phase II, a pilot RCT was conducted to evaluate the feasibility, acceptability and implementation of this intervention among participants with cognitive impairment.

## Methods

Reporting of this feasibility study was guided by the Consolidated Standards of Reporting Trials extension to randomised pilot and feasibility trials guidelines [42]. The Office for Research Ethics Committees Northern Ireland (ORECNI) and Belfast Health and Social Care Trust Research Governance provided ethical approval for the study, and the study was registered via ClinicalTrials.gov (NCT03265522; NCT03569319). This paper has been presented as the pilot RCT, with the qualitative work embedded in the development phase.

### Participants and recruitment

Participants were recruited (to Phase II) from memory clinics within the Belfast Health and Social Care Trust, Northern Ireland (NI). Recruitment was facilitated by Geriatricians and Psychiatrists who identified potentially eligible participants from their clinics and distributed information leaflets. Participants who agreed for a follow-up telephone call were contacted by the study researcher (AMG). Enrolment was based on the following criteria: Inclusion: (1) Diagnosis of MCI by their physician and according to NIA-AA criteria [43], (2) Mediterranean Diet Score (MDS) ≤ 4 points as measured by validated questionnaire adapted from the PREDIMED study to suit a Northern Ireland population [44, 45] and (3) Willingness to make changes to their diet. Exclusion: (1) Diagnosis of dementia; (2) Patients with a visual or English language impairment; (3) Psychiatric problems; (4) Significant medical comorbidity; (5) Body mass index (BMI) ≤ 19 and ≥ 40 kg/m^2^; (6) Excessive alcohol consumption; (7) Dietary restrictions/allergies that would substantially limit ability to complete study requirements; (8) Taking high dose nutritional supplements (supplements above Reference Nutrient Intake levels); (9) Inability to provide informed consent; (10) History of, or comorbid condition, which may alter performance on cognitive tests, e.g. stroke, head injury, Parkinson’s disease, learning disabilities. Potentially eligible participants were invited to take part in a screening appointment, either in their own home or within the research facilities in the Centre for Public Health at Queen’s University, with the study researcher (AMG/SM). At this appointment, participants completed a MDS and medical screening questionnaire, as well as had measurements of height, weight and blood pressure recorded.

### Dietary intervention

#### Intervention selection and development

**Fig. 1:**
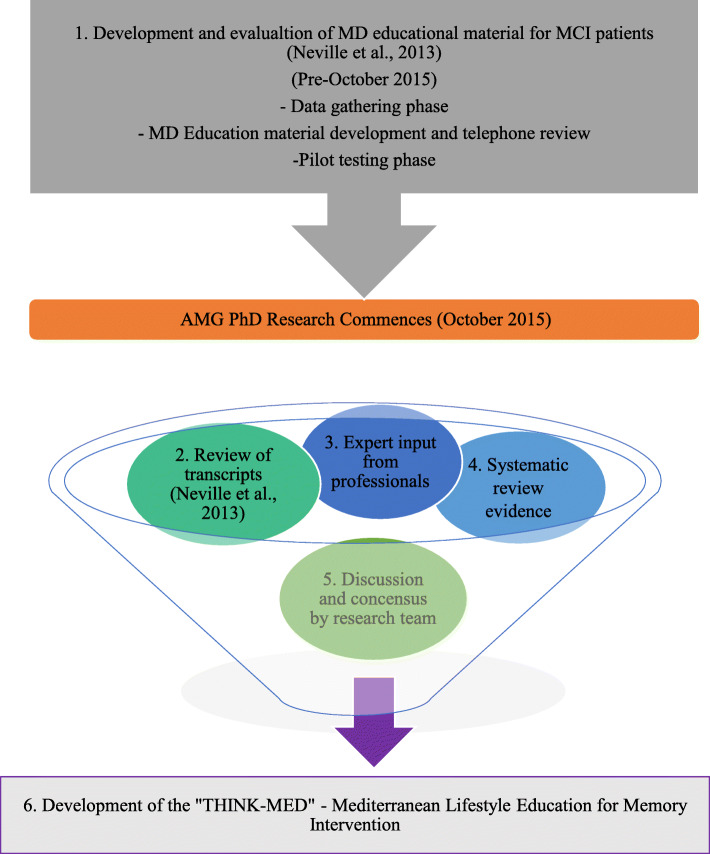
Sources of information used to refine the MD educational material (Neville et al. 2013) and to develop the THINK-MED intervention

The flow of work for the development of the THINK-MED intervention is presented in Fig. 1. Qualitative research conducted by Neville et al. (2013) evaluated the usability and comprehensibility of MD educational material to encourage dietary behaviour change among *n* = 21 MCI patients and their carers (*n* = 6) (*Step 1*). The results of this study have been published elsewhere [41]. The transcripts and telephone questionnaires from the study by Neville et al. (2013) [41] were reviewed to highlight the feedback received from MCI patients and their care givers in order to ensure these aspects informed refinement of this educational material (*Step 2*). The transcripts were analysed deductively in order to improve the materials and develop a tailored dietary intervention. Within the educational material, MCI patients wanted more information on memory, specifically the memory and diet link, physical activity recommendations as well as inclusion of prompts or reminders to support dietary changes. Furthermore, a staged approach in the delivery of educational materials was deemed most useful to support MCI patients in learning and retaining new information over a period of time. Based on this feedback, it was agreed by the research team that a pilot study was necessary, in order to understand the level of support needed for this patient group to make changes towards a MD (with or without a dietitian) and to determine if the information provided influenced MD and lifestyle behaviour change (complete resource on one occasion vs staged delivery).

In order to adopt a lifestyle approach to the intervention and as requested via qualitative feedback by Neville et al., experts in physical activity for older adults with cognitive impairment were consulted to obtain guidance on appropriate exercise information for the target group. An occupational therapist advised on cognitive strategies, e.g. reminders and memory aids, that are suitable for patients with MCI (*Step 3*). A systematic review of dietary interventions in MCI patients [40] was conducted as the research team hoped to identify any completed MD intervention studies that had been conducted in this population group to inform the design of the present research (*Step 4*). The literature search identified that there had been no MD intervention studies completed among MCI patients. Ultimately, this confirmed a need for such novel research and supported the rationale for the implementation of this intervention. The research team (JW, BMG, MMK, CME and AMG) discussed the information gathered from the Steps 1–4 to reach consensus on the refinements to be made to the MD educational material and intervention design (*5*).

#### THINK-MED intervention description

The THINK-MED - Mediterranean Diet and Lifestyle Education resource that was evaluated by MCI patients (Phase I) consisted of the following: (1) Mediterranean Diet and Lifestyle Education for Memory Information Booklet, (2) a calendar containing recipes and tips, (3) Memory Book which focused on goal setting and memory aids, e.g. shopping lists, (4) Seasonal Recipe Books and (5) 7-day MD Menu Plans. A goal setting approach was included to support the dietary information provided, focusing on five key MD goals (to increase fruit and vegetables, olive oil, natural nuts, oily fish and wholegrains). For each key dietary goal, information was provided within the Information Booklet on the benefits in terms of memory, tips on what the participant could do to increase intake, an explanation of common barriers to increasing intake with suggestions on how to overcome these. Further instructions on how to set a dietary goal as well as templates to record weekly goals and track progress were included in the Memory Book, together with practical examples. An outline of the intervention materials can be found in the supplementary material. It was proposed that in a pilot RCT participants would be randomised into one of three groups: (1) THINK-MED - Mediterranean Diet and Lifestyle Education for Memory resource at baseline (complete resource provided on one occasion), (2) THINK-MED - Mediterranean Diet and Lifestyle Education for Memory resource staged over a period of time (with support from a dietitian at an initial face-to-face consultation and monthly telephone reviews to discuss dietary goals) and (3) control (delayed intervention).

#### Qualitative evaluation (Phase I)

The THINK-MED resources were evaluated by MCI patients via structured interviews to evaluate their acceptability and to explore the perceived barriers and enablers to dietary change. MCI patients were recruited from memory clinics in the Belfast Health and Social Care Trust. Participants had a diagnosis of MCI determined by their physician based on the NIA-AA criteria [43] and were interested in learning about a MD and lifestyle. Suitable participants were identified and informed of the study by their physician. If the MCI participant was not responsible for food provision in the household, relatives who were responsible were also invited to participate in the interview. Interested participants were contacted by the researcher (AMG) and if agreeable to take part, the ‘THINK-MED’ - Mediterranean Lifestyle Education resource was posted to each participant at least 1 week before their scheduled interview for consideration and appraisal.

The Theoretical Domains Framework (TDF), a theory-driven behavioural framework that can aid the design and development of complex interventions, was used to structure the interview schedule and, in the analysis, as an initial coding framework. The framework was then expanded to reflect the emerging themes from the study [46, 47]. The main areas covered in the topic guide are listed in an additional file (Supplementary Material Table 1). With participant consent, the interviews were audio-recorded and transcribed verbatim. AMG collected and analysed all the qualitative data.

A total of eight MCI patients took part in the structured interviews (mean age 77.0 years); two participants were accompanied by a family member. Feedback from the qualitative discussions revealed that the material was acceptable to MCI patients and each participant had made some changes towards a MD, or intended to, after reading the resource. Participants were optimistic with respect to making dietary changes and the idea that a MD could improve their health, particularly their memory, was emphasised as an enabler to dietary change. Understandably, one of the main barriers for this group were the effects of MCI on their ‘memory, attention and decision processes’, such as ability to retain information and need for reminders. There were practical suggestions made by participants to improve the format and content of the resource, to include clarification of portion sizes, provision of simpler meal ideas with smaller serving sizes to suit the needs of an older adult population as well as more structure to guide the reader through the various parts of the resource. Although participants had the intention of making dietary changes, there were indications that the proposed method for goal setting may not suit everyone, as it was suggested by some to be too prescriptive and time-consuming to record goals in a written format. Table 1 provides an overview of representative participant quotes from the qualitative interviews. The feedback gathered in this phase was used to make further refinements to the proposed Mediterranean Diet and Lifestyle Education intervention, THINK-MED, before its evaluation through a pilot RCT (Phase II).

**Table 1:** Summary table of MCI participant interview quotes matched to TDF domain

TDF domain (Michie et al. 2014)	COM-B component	Sub-theme	MCI Participant Interview Representative Quotations (All patient quotes unless otherwise stated)
**Knowledge**	Capability (psychological)	Mediterranean diet knowledge	“I had no clear knowledge that there was an association with memory” “I never thought about diet helping memory but I was aware that diets were important in other things” “Well we knew that the Mediterranean diet was so healthy, but we didn’t know it was significant in memory” (relative of participant 008) “I’ve heard of the Mediterranean diet as being a very healthy diet”
**Skills**	Capability (physical)	Cooking Current diet	“Maybe a lot of people in mid 80s don’t do a lot of cooking” “Well my wife for instance, years gone by you would have cooked with lard or something like that, but now would be oils like olive oil or rapeseed oil” “We eat, my husband and I, both make sure we have vegetables, make sure we have fruit, it’s the in-between things that we eat are not so good”
**Optimism**	Motivation (reflective)	Perceived benefits and motivation to change	“I really want to give it a go, for two reasons, for the memory and also it is healthy eating … I’ve put on a lot of weight... so I was hoping that this healthy eating will be able to decrease that” “The people who have this kind of diet don’t have the same memory loss as early” “The payoff is obviously magnificent because if it retards or reduces memory loss then it’s a positive” “I’m not going to have turkey and bean mash for my Christmas dinner... Now, saying I wouldn’t but if I was told my life depended on it, I would do it … ” “It would all depend on whether I thought it was going to be something that was good for me... the main thing is that you must want to do it” “I know things like that, that things would be better, I would still be careless, I know I would be” “If it helps, I definitely would try it” “I am prepared to try these”
**Environmental context and resources**	Opportunity (physical)	Evaluation of the THINK-MED resource THINK-MED calendar THINK-MED recipes	“It’s attractive... it’s an attractive booklet … it just appealed … Probably with the pictures, print everything”, “It was very attractive, I thought it was very informative” (relative of participant 002) “Clear and easy to understand” “I thought there was a good introduction on mild cognitive impairment which I wasn’t really totally familiar with, what it implied” “I thought that was an interesting diagram too (MD pyramid)” “Reading those with diagrams on it is much easier and it tells you how much you should eat of those meals” “I had a rough idea about it, and then from reading your information, put more of a shape to it … and took away a lot of the hesitancy I had about it” “I think it’s an interesting diet and the resource is colourful” “The word wholegrain … I don’t think everybody would necessarily know what that term means” “Perhaps it would help if they were numbered or identified in some way that people would know which one to read first … they were probably in the order that you wanted them to be looked at, but once you took them out they got mixed up” “I would be thinking where that is? Which menu or book is that in to find that, to prepare it?” “The recipes that you have each month, I’m not sure, wasn’t sure whether they were suggested recipes for this particular month for any reason?” “you can write down things, something that you are going to do, maybe something that you have done that day” “Some of the recipes are impractical … like ourselves, when you’re pensioners and that, would have a smallish lunch, but it wouldn’t be anything very elaborate that would take long to cook or prepare” “Olive oil or rapeseed oil, she thought the amounts were too much … she thinks that some of the recipes are impractical, and unlikely to be used (wife)” “Yes some of them looked very attractive...”, “I think you could use them as a base... and then maybe change some ingredients to suit what we would like, like turkey and bean mash, we wouldn’t be fans of turkey but could change it to chicken” (relative of participant 002) “And they are not too difficult, well I mean at least I don’t think they are too difficult... Which is good because I like really simple recipes although my husband does the cooking I am starting to do more and more … ” (relative of participant 008)
**Goals**	Motivation (reflective)	Goal setting	“The goal setting was quite interesting and perhaps a useful thing for people to attempt … the idea of breaking it down in to sort of small steps” “I don’t think, I would sit down and make a list of dietary goals and try and achieve them” “I don’t think I would participate in the study, because I wouldn’t be bothered, doing all that” “I think you would have to do that otherwise you wouldn’t stick to it … but if you have it written down, I think you are more inclined to stick to it” (relative of participant 002) “I really can’t see me doing that, I think that would be the right thing to do, but I just can’t see me doing that” “I would be inclined now to write it down … as I say about the memory loss … one thing I’ve started doing now, is I put down dates”
**Intentions**	Motivation (reflective)	Encouraged to change Not everyday	“I think it would probably encourage us to make a few additional changes that perhaps we haven’t made before … I think my wife will look at the recipes and maybe pick out, one or two that would suit us” “we have resolved independently that I don’t want to eat steak again” “I’ve changed to my brown rice and I am using up my ordinary pasta first and will change that” “well I have already changed to wholegrain bread … we would have had red meat regularly, now cut that out quite a good bit” “I’m not going to have turkey and bean mash for my Christmas dinner” “Probably a Mediterranean diet wouldn’t appeal to me on a Sunday … I probably wouldn’t want to do it every day and so on and so forth”
**Beliefs about capabilities**	Motivation (reflective)	Delivery of information Challenges: cost Challenges: support Challenges: age Challenges: enthusiasm	“Maybe there are others who would need a bit of help or support throughout but I find it very simple to understand … I’m happy just doing it myself” “My first reaction was shock when I saw how much information there was … when I opened it up I realised it was quite well spaced out and it wasn’t just quite as much information as I first anticipated” “it was too much all at once … overwhelming … all I would have liked would have been less of it” “Well I know sometimes there’s hints about the price and maybe if you couldn’t afford the stuff and the fresh vegetables and fruit” “Or maybe they think it is more expensive to go and buy all the ingredients” (Relative of participant 002) “well expense would for a lot of pensioners, it would put them off … so now you have to watch the purse as well, like olive oil is dearer than other oils” “I would still be careless, I know I would be, so I would have to rely on my son from now on a bit … when he last visited, he had a look at this, and he will probably ask me have you done this?” “Well I think I would be very happy because my wife and I are doing it together… which is a way of support and in doing that we can work out the menus” “when you get to 80, you’re more … just whatever comes to your head at any moment” “My problem might be that I will lose enthusiasm”
**Reinforcement**	Motivation (automatic)	Reminders	“reminders … just to remind you, maybe that wee pyramid so like a - remember this - type thing” (relative of participant 002) “especially when your memory is not that good, it would maybe trigger something off “using a diary in my pocket and writing down what I’ve got to do during the day and refer to that then because it’s something I can keep on myself and if I get caught during the day I can pull it out”
**Memory, attention and decision processes**	Capability (psychological)	Effects of MCI	“Now and again my memory just lapses, you know during talking about something I would go along and suddenly my memory would drop, off the face of the earth … ” “I find that I need to write it all down, write it out again … so that I would write out breakfasts, lunches and I know that they are in here … because if I read it last night, what I am going to have for my breakfast, that’s gone” “well I’ve a feeling that my memory isn’t as good, in fact I’m sure it’s not, I just see a deterioration” “what I find at the moment is that in the morning I have to refer or ask my wife what’s happening today, once that’s done, I don’t forget about it” “I think the thing that frightens people and frightens me as well so it does is the thought of constant and deepening memory loss and one would like to try and fight that” “I would say my brain has got duller since I have been sitting in the house on my own … no I don’t write it out … I like to go by the picture and go back and think of what I did”
**Behavioural regulation**	Capability (psychological)	Planning	“The meal planning and shopping lists, would people actually use them? I suppose some people would and others wouldn’t” “yea something to work towards … just looking at it rather than thinking what will I have to eat … think that is a good idea, it just takes a bit of preparation, I think it would be better to plan ahead, try and plan for a week”

#### Refinement of Mediterranean Diet and Lifestyle Education resource THINK-MED before pilot testing

The calendar was not included in the final educational resource as it was suggested to have no additional benefit and was confusing for participants to follow. To provide more structure, a contents list was inserted into the resource, as well as being numbered to guide the reader. Further examples of simpler MD meal options were added to the THINK-MED Information Booklet, with clarification of portion sizes where necessary. Within each recipe book, messages were reinforced to indicate that the recipes could be adapted to suit individual tastes and the ingredients could be varied accordingly in order to make the recipes less restrictive. With regard to goal setting, upon refinement, this strategy was promoted as an option for participants to use, explaining how to do this effectively through the use of examples and condensing this information into a more user friendly and attractive format. This would enable participants to use these as reminders if they so wish, to reinforce the implementation of the MD. Thus, the final THINK-MED - Mediterranean Diet and Lifestyle Education for Memory resource included (1) THINK-MED Information Booklet, (2) Four Seasonal Recipe Books, (3) THINK-MED Menu Plan Cards, (4) THINK-MED Shopping List Cards and (5) THINK-MED Goal Setting Cards. A copy of the final THINK-MED resources can be found in the supplementary material. There were suggestions from the qualitative feedback that smaller amounts of information may be more manageable for some individuals to make dietary changes. Therefore, to understand the level of support needed for this patient group to make changes towards a MD (with or without a dietitian), and to determine whether the delivery of information has an influence on MD and lifestyle behaviour change (complete resource on one occasion vs staged delivery), the proposed THINK-MED intervention to be pilot tested would involve the following:

1. THINK-MED Mediterranean Diet and Lifestyle Education for Memory resource provided on one occasion
2. THINK-MED Mediterranean Diet and Lifestyle Education for Memory resource staged over 5 months, accompanied by an initial face-to-face meeting with a dietitian (with the study participant and their spouse or relative (if the spouse/relative was responsible for food provision)) followed by telephone reviews each month to discuss dietary goals
3. Control (delayed intervention) – participants received the complete THINK-MED Mediterranean Diet and Lifestyle Education for Memory resource following completion of their final study visit.

This intervention design remained unchanged from the proposed intervention before qualitative evaluation by MCI participants.

### Outcome assessments

The primary outcome of this pilot RCT was a change in MDS at 6 months, with a maintenance of score by 12 months. All of the outcome measurements collected are detailed in Table 2, and these were intended to gather data on variability of the outcomes to inform a full trial power calculation and to test acceptability of the measures. Participants enrolled in the THINK-MED study were asked to complete outcome assessments at three time points – baseline, 6 months and 12 months. Participants were visited in their own homes or convenient location by the study researcher and outcome assessments were conducted. These visits lasted approximately 3.5 hours to allow the following measurements to be taken: a fasting blood sample, cognitive testing, anthropometric measurements, blood pressure, grip strength and completion of the various dietary, lifestyle, behaviour change and psychological questionnaires. Home visits were scheduled in the morning to facilitate the fasting blood sample and arranged at a pre-agreed time. At the final study visit, participants were also asked to complete a study evaluation questionnaire to appraise adherence to the intervention as well as their overall experience of participating in the study.

**Table 2:** Outcome measures for THINK-MED pilot RCT

Outcome measure	Description of measure used
**Primary Outcome**	
Mediterranean Diet Score [44]	A change in MDS at 6 months, with a maintenance of score by 12 months. This 14-item MDS was adapted from the validated MDS used in the PREDIMED study [45] for use among Northern European populations. The overall aim was to increase MDS by ≥ 3 points at month 6 (adoption) and month 12 (maintenance) (if applicable). The questionnaire contained 14 items based on consumption of a component of the MD. Each item was scored either zero or one. The maximum score which could be obtained was 14, with a score of zero indicating a low adherence to a MD and 14 indicating the greatest adherence.
**Feasibility considerations**	
Feasibility	**Recruitment** (recruitment rates**,** time taken for the recruitment of participants**,** characteristics of recruited sample as against expected, non-response rates, reasons for non-response) **Retention and completion** (retention rates**,** number of drop outs and reasons for withdrawal, follow-up response rates (6 months and 12 months of follow-ups) **Data collection methods and study procedures** (Feasibility of measurement tools **-** time taken to fill in questionnaires, missing data from questionnaires, understanding level of procedures and measurement protocols**,** suitability of outcome measures, sensitivity of outcome measures to change) **Acceptability and adherence to intervention** (barriers to participation, burden (reasons for not taking part/discontinuation or dropping out, study evaluation questionnaire))
**Outcomes to be tested for a definitive trial**	
Cognitive function	A change in cognitive test score at 6 months, with a maintenance of score by 12 months. A comprehensive cognitive assessment was performed using a validated neuropsychological test battery provided by Cambridge Cognition Ltd. (CANTAB). This involved seven tests specific and sensitive to a MCI population. Tests included: Motor Screening Task (MOT), Reaction Time (RTI), Paired Associates Learning (PAL), Pattern Recognition Memory (PRM), Delayed Matching to Sample (DMS), Spatial Working Memory (SWM) and Rapid Visual Information Processing (RVP).
Physical activity	RPAQ-Recent Physical Activity Questionnaire [48]
Anthropometry	Height (m), Weight (kg), BMI (kg/m^2^), waist circumference (cm), hip circumference (cm), waist to hip ratio
Muscle strength	Measured by a hand grip-strength dynamometer
Blood pressure	Branchial blood pressure was obtained using a calibrated Omron automated monitor (British Hypertension Society approved).
Demographic and medical history	Demographic, lifestyle and medical questionnaire (baseline) and change in lifestyle questionnaire (6/12 months)
Instrumental activities of daily living (IADL)	IADL scale by Lawton and Brody (1969) [49] and Bristol activities of daily living (ADL) scale (Bucks et al. 1996) [50]
Depression	Geriatric Depression Scale (GDS) (short form) [51]
Quality of life	Rand 36-item validated questionnaire [52]
Health beliefs	Previously validated questionnaire based on components of the Health Belief Model [53] which was modified for use within the TEAM-MED study [44]
Dietary behaviour change	MD staging algorithm [54] MD knowledge (adapted from Parmenter and Wardle, 1999) [55] Barriers to MD dietary change (based a questionnaire devised from on previous focus group analyses with a Northern European population used within the TEAM-MED study [44]) MD self-efficacy [56] 4 day food diary to capture dietary intake and measure MD compliance
Study evaluation	Data collected via questionnaire on evaluation of study procedures and tolerance towards a MD
Blood sample	50 ml blood sample was taken from all participants to be stored for future analysis of biomarkers of MD adherence and biomarkers of neurodegenerative disease risk

### Randomisation

Following screening, participants were randomised, using a block design, to one of three groups. Each participant was given a three-digit study number. The randomisation sequence was computer generated (http://www.randomization.com) by a member of the research team (JW) who was not involved in the data collection.

### Sample size

No formal sample size calculation was performed for this pilot RCT, as the aim was to assess feasibility. Any investigations of changes in key study parameters were exploratory only. A sample size of 60 was based on what was considered a feasible recruitment target within the given timeframe through discussions by the research team and physicians as well as based on the estimated effect size provided by the guidelines by Whitehead et al. [57]. Specifically, a sample size of 20 individuals per group would allow population variance to be estimated and allow estimation of key parameters for a full trial.

### THINK-MED protocol changes

Due to slower recruitment rates than anticipated (discussed later), the research team made an informed decision to submit an amendment to the research ethics committees to reduce the intervention period from 12 months to 6 months for any eligible participants recruited after the 1st of April 2018. This allowed recruitment to be extended by a further 6 months and provide an opportunity to boost the study sample. Thus, all participants enrolled in the THINK-MED study had outcome measurements taken at baseline and 6 months, with an additional 3rd visit at 12 months for those who were enrolled before the 1st of April 2018. An additional sub-study was also conducted among community-dwelling participants with subjective cognitive impairment (SCI) using the same THINK-MED dietary intervention and data collection measures (NCT03569319), in an attempt to increase recruitment capacity and to determine the feasibility of recruitment from a non-clinical population. These individuals were recruited over a period of 3 months by poster placement and group visits facilitated by older adult charities in NI. The study advertisement materials (see Supplementary Material) were disseminated to the local community via charitable groups and organised educational outreach activities. At these activities, a study researcher (AMG) promoted the study through group discussions with interested participants. Inclusion and exclusion criteria for this study remained the same as stated previously; however, SCI was determined by the Montreal Cognitive Assessment (MoCA) [58], whereby a score of 26 or above was used for participant inclusion. Thus, potentially eligible participants were invited to take part in a screening appointment, either in their own home or within the research facilities in the Centre for Public Health at Queen’s University, with the study researcher (AMG/SM). At this appointment, participants completed a MDS, MoCA and medical screening questionnaire, as well as had measurements of height, weight and blood pressure recorded. Finally, these recruitment challenges necessitated a change from the originally intended primary outcome of a change in MDS as stated on the trial registration document (Clinical trials registration NCT03265522/NCT03569319) to a revised focus primarily on feasibility.

### Analysis

Descriptive statistics were used to summarise the screening, eligibility, randomisation, adverse events, retention, completion, missing data and intervention adherence rates. Feasibility considerations are described in a narrative format, with tables and figures where appropriate. Feasibility was considered in terms of the ability to recruit and retain participants and implementation of the THINK-MED intervention. In addition, evaluation of the study procedures and intervention engagement was assessed post-intervention via a self-administered evaluation questionnaire as well a review of the researcher field notes. Demographic data are presented as means and standard deviations or frequencies and/or percentages where appropriate. In this paper, we also report on the MDS data. Cognitive assessment data is available in an additional file (Supplementary Material Tables 2 and 3). Owing to the small study sample and feasibility nature of this research, these data are presented descriptively.

## Results

As the results of Phase I have already been summarised and were used to inform and develop the intervention for the pilot study, the results section of this manuscript is focused on the results of Phase II - THINK-MED pilot RCT.

### Study characteristics

**Fig. 2:**
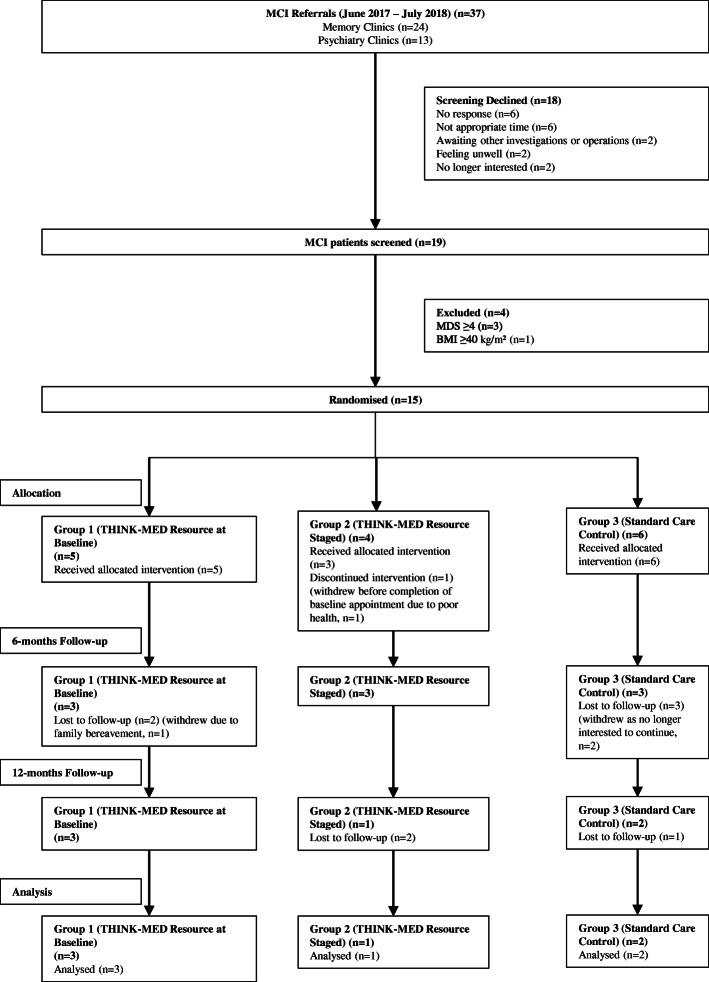
CONSORT diagram summarizing flow of MCI participants through the THINK-MED study

**Fig. 3:**
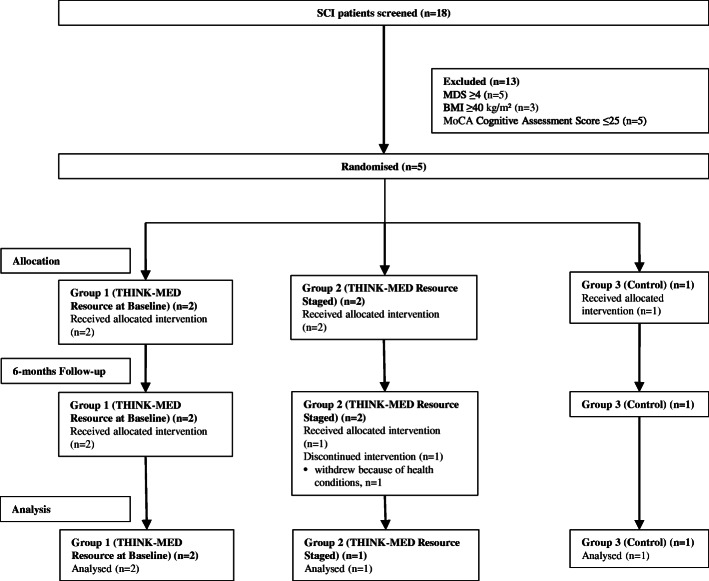
CONSORT diagram summarizing flow of SCI participants through the THINK-MED study

There were a total of 20 participants (*n* = 15 with MCI and *n* = 5 with SCI) enrolled in the THINK-MED study and the flow of participants can be seen in Figs. 2 and 3. The baseline characteristics of MCI study participants are presented in Table 3. The sample included a total of 14 participants (*n* = 1 withdrew before baseline appointment), 9 (64 %) males and 5 (36 %) females. The mean age of participants was 76.5 ± 7.0 years. None of the participants were smokers and 36% consumed alcohol. The mean years spent in full-time education was 14.4 ± 4.0 years. Over half of the participants (57%) were married, 29% divorced and 7% were single or co-habiting. At baseline, the mean MDS was 2.8 ± 1.2, indicating that participants had a relatively low adherence to a MD at the start of the study. The participants in the THINK-MED Staged’ group had the lowest mean MDS (1.3 ± 1.2) compared to participants in the other groups. The baseline characteristics of the THINK-MED SCI study participants are presented in Table 3. The sample included 5 participants, 2 (40%) males and 3 (60%) females with a mean age of 67.2 ± 7.9 years. None of the participants were smokers, and 60% of them consumed alcohol. The mean years spent in full-time education was 12.2 ± 2.9. The participants were married (40%), divorced (40%) and single (20%). At baseline, the mean MDS was 3.8 ± 2.8, indicating that participants had a relatively low adherence to a MD at the beginning of the study. The participants in the ‘control’ group had a high mean MDS (8.0 ± 0.0) compared to participants in other two groups. For this group, it is important to note that 1 participant recorded a MDS of 4.0 at screening, and upon baseline assessment, their score had increased to 8.0. This is likely to explain the high mean MDS value for the control.

**Table 3:** Baseline characteristics of THINK-MED MCI and SCI participants

	MCI (***n*** = 14)								SCI (n = 5)							
	THINK-MED participants (***n*** = 14)		Group 1: THINK-MED at baseline (***n*** = 5)		Group 2: THINK-MED staged (***n*** = 3)		Group 3: Control (***n*** = 6)		THINK-MED participants (***n*** = 5)		Group 1: THINK-MED resource at baseline (***n*** = 2)		Group 2: THINK-MED resource staged (***n*** = 2)		Group 3: Control (***n*** = 1)	
Characteristics	Mean (SD)	Range	Mean (SD)	Range	Mean (SD)	Range	Mean (SD)	Range	Mean (SD)	Range	Mean (SD)	Range	Mean (SD)	Range	Mean (SD)	Range
Age (years)	76.5 (7.0)	60.0–86.0	79.2 (5.0)	73.0–86.0	73.7 (12.1)	60.0–83.0	75.7 (6.0)	67.0–84.0	67.2 (7.9)	60.0–78.0	62.5 (0.7)	62.0–63.0	66.5 (9.1)	60.0–73.0	78	–
Males, *n* (%)	9 (64.3)		4 (26.7)		3 (20.0)		2 (13.3)		2 (40.0)		0 (0.0)		2 (40.0)		0 (0.0)	
BMI (kg/m^2^)	27.9 (3.8)	22.7–35.6	26.0 (3.2)	22.7–30.5	28.6 (4.8)	25.0–34.0	29.1 (4.1)	25.0–35.6	30.4 (2.9)	28.4–35.1	32.1 (4.2)	29.1–35.1	28.5 (0.2)	28.4–28.3	31	–
Normal, *n* (%)	4 (28.6)		2 (14.3)		0 (0.0)		2 (14.3)									
Overweight, *n* (%)	6 (42.9)		2 (14.3)		2 (14.3)		2 (14.3)		3 (60.0)		1 (20.0)		2 (40.0)		0 (0.0)	
Obese, *n* (%)	4 (28.6)		1 (7.1)		1 (7.1)		2 (14.3)		2 (40.0)		1 (20.0)		0 (0.0)		1 (20.0)	
Waist hip ratio	0.93 (0.10)	0.90–1.00	0.96 (0.05)	0.90–1.03	0.95 (0.04)	0.91–0.98	0.90 (0.05)	0.85–0.99	0.96 (0.05)	0.91–1.03	0.95 (0.04)	0.92–0.98	0.99 (0.05)	0.96–1.03	0.91	–
Current smoker, *n* (%)	0 (0.0)		0 (0.0)		0 (0.0)		0 (0.0)		0 (0.0)		0 (0.0)		0 (0.0)		0 (0.0)	
Previous smoker, *n* (%)	9 (64.3)		4 (28.6)		2 (14.3)		3 (21.4)		3 (60.0)		0 (0.0)		2 (40.0)		1 (20.0)	
Alcohol consumer, *n* (%)	5 (35.6)		4 (28.5)		0 (0.0)		1 (7.1)		3 (60.0)		1 (20.0)		2 (40.0)		0 (0.0)	
Systolic blood pressure (mmHg)	147.3 (21.2)	92.0–173.5	149.6 (13.7)	141.0–173.5	146.6 (23.6)	122.0–169.0	145.8 (28.0)	92.0–164.0	141.1 (13.8)	130.0–159.0	131.8 (1.1)	131.0–132.5	141.5 (16.2)	130.0–153.0	159	–
Diastolic blood pressure (mmHg)	80.2 (17.8)	50.0–10.9	79.0 (19.4)	53.0–105.0	79.7 (15.3)	62.0–89.0	81.5 (20.7)	50.0–109.0	83.9 (19.0)	62.0–111.5	78.0 (9.8)	71.0–85.0	100.8 (15.2)	90.0–111.5	62	–
Grip strength (mm)																
Left arm	25.2 (9.4)	12.0–44.3	26.2 (5.3)	19.2–30.7	33.6 (9.5)	26.0–44.3	20.9 (10.2)	12.0–38.0	20.9 (14.2)	5.0–40.7	15.0 (14.1)	5.0–25.0	32.5 (11.6)	24.3–40.7	9.3	
Right arm	27.5 (10.4)	11.0–50.0	27.9 (6.8)	19.7–35.5	37.4 (11.6)	27.2–50.0	27.2 (9.8)	11.0–38.0	24.7 (12.7)	9.7–41.5	20.7 (8.5)	14.7–26.7	36.1 (7.6)	30.7–41.5	9.7	
Nutritional supplements, *n* (%)	9 (64.3)		4 (28.6)		2 (14.3)		3 (21.4)		1 (20.0)		1 (20.0)		0 (0.0)		0 (0.0)	
Souvenaid, *n* (%)	2 (22.2)		1 (11.1)		1 (11.1)		0 (0.0)		0 (0.0)		0 (0.0)		0 (0.0)		0 (0.0)	
Years in full-time education (years)	14.4 (4.0)	10.0–20.0	15.1 (4.2)	11.0–20.0	11.3 (2.3)	10.0–14.0	15.3 (4.3)	11.0–22.0	12.2 (2.9)	8.0–16.0	12.5 (0.7)	12.0–13.0	14.0 (2.8)	12.0–16.0	8	–
Relationship status, *n* (%)																
Single	1 (7.1)		0 (0.0)		0 (0.0)		1 (7.1)		1 (20.0)		1 (20.0)		0 (0.0)		0 (0.0)	
Co-inhabiting	1 (7.1)		1 (7.1)		0 (0.0)		0 (0.0)		0 (0.0)		0 (0.0)		0 (0.0)		0 (0.0)	
Married	8 (57.1)		3 (21.4)		2 (14.3)		3 (21.4)		2 (40.0)		0 (0.0)		1 (20.0)		1 (20.0)	
Divorced	4 (28.6)		1 (7.1)		1 (7.1)		2 (14.3)		2 (40.0)		1 (20.0)		1 (20.0)		0 (0.0)	
Widowed	0 (0.0)		0 (0.0)		0 (0.0)		0 (0.0)		0 (0.0)		0 (0.0)		0 (0.0)		0 (0.0)	
MDS	2.8 (1.2)	0.0–4.0	3.4 (0.5)	3.0–4.0	1.3 (1.2)	0.0–2.0	3.0 (1.1)	2.0–4.0	3.8 (2.8)	-	2.5 (0.7)	2.0–3.0	3.0	1.0–5.0	8.0	–

### Feasibility considerations

#### Findings related to recruitment

A total of 20 participants (*n* = 15 MCI and *n* = 5 SCI) were enrolled in the THINK-MED study, a third of the anticipated target sample size (*n* = 60). MCI recruitment commenced in June 2017 (first participant enrolled in August 2017) and ended in July 2018, with *n* = 37 referrals from geriatrician/psychiatrist led clinics during this timeframe. Recruitment of MCI patients from the clinical setting was challenging owing to a number of factors. Feedback from physicians suggested the main challenges in referring patients were (1) inappropriate referrals to memory clinics from primary care services and thus not having a diagnosis of MCI upon investigation, (2) difficulties in remembering to promote the study to patients due to busy caseloads and (3) competing demands from a number of MCI research studies that were running alongside each other. Upon contact by the study researcher, a total of 18 (49%) participants declined a screening appointment, with 6 (16%) indicating that it was not an appropriate time for them to take part in a research study due to a variety of reasons such as recent family bereavement, family commitments or busy lifestyles. Other reasons for screening decline included feeling unwell and no longer keen to take part (*n* = 2, 5%), awaiting other medical investigations, such as an MRI scan or a hip replacement operation (*n* = 2, 5%), no longer being interested in the study (*n* = 2, 5%) or failure to respond to the researchers phone calls/voicemails/letters (*n* = 6, 16%). Of the 19 (51%) participants who were screened for eligibility, 3 were ineligible as they obtained a MDS greater than 4 points and 1 had a BMI greater than 40 kg/m^2^.

For SCI participants, although the final numbers enrolled in the THINK-MED study were small (*n* = 5), it is clear that recruitment through a community-dwelling population using charitable organisations had more reach, informing a larger number of people about the research (*n* = 41) compared to the number of MCI referrals (*n* = 37) made during a 1-year period. The limited time frame for recruitment for the THINK-MED SCI study (3–4 months; June 2018–September 2018) was the main challenge to recruitment, given that it was initiated when it became apparent that recruitment from the MCI group was lower than anticipated. However, being able to source and inform 41 participants about the THINK-MED SCI study in this short period suggests that there was potential for this number to be amplified if the recruitment timeframe could have been extended. Furthermore, as the timing of the recruitment took place over the summer months, many community groups were on a summer break and not scheduled to resume until September. We also found that one particular charity seemed to work with individuals who were much older and dependent on the charity’s services (e.g. some lived in nursing homes or had poor functionality) compared to those from other organisations who were more active in the community, many of whom were group leaders or organisers. This therefore limited the number of people who met the inclusion criteria for the study from this organisation.

#### Findings related to inclusion/exclusion criteria

As the original recruitment target of *n* = 60 for the THINK-MED study was not met, merely achieving a third of this target (*n* = 20), it could be suggested that, together with the recruitment challenges discussed, some of the eligibility criteria for the THINK-MED study may have been too stringent.

##### MDS (≤ 4 points)

For inclusion in the THINK-MED study, participants had to obtain a MDS of less than or equal to 4 points using a validated questionnaire [44]. 3 MCI and 5 SCI participants were deemed ineligible as they had a MDS of > 4 points. This cutoff for inclusion for the THINK-MED study was chosen as it was feasible for a similar NI-based MD intervention, TEAM-MED [44].

##### Nutritional supplements

It became apparent upon screening MCI patients that some were consuming the medical food, Souvenaid, as recommended by their physician. Souvenaid is a multi-nutrient supplement drink containing a combination of DHA and EPA fatty acids; uridine monophosphate; choline; vitamins B12, B6, C and E; and folic acid, phospholipids and selenium [59]. Furthermore, 7 (63 %) participants enrolled in the THINK-MED study were consuming other nutritional supplements, such as omega 3 fatty acids (*n* = 1, 9%), folic acid (*n* = 3, 27%), vitamin C (*n* = 1, 9%), iron (*n* = 1, 9%) and vitamin D (*n* = 1, 9%). Thus, the team agreed that participants who were taking nutritional supplements would be eligible to take part, as long as they satisfied all other inclusion criteria, and this was recorded on the participant’s file.

##### MoCA cognitive assessment (SCI study only)

In order to determine SCI, an additional screening component was added involving cognitive assessment using the MoCA questionnaire. In order to be eligible, participants had to score within the normal range for age related cognitive decline (SCI) - ≥ 26 points. Based on this, 5 participants (12%) were not eligible for inclusion in the THINK-MED SCI study due to a low MoCA score, thus indicative of potential objective impairment.

##### BMI

Finally, 3 (7%) SCI and 1 (5%) MCI participants were not eligible for the THINK-MED study as they had a BMI above the exclusion criteria of ≥ 40 kg/m^2^. This criteria was included due to potential difficulties with obtaining a blood sample from adults with a BMI ≥ 40 kg/m^2^ in a non-clinical setting.

#### Findings related to retention and adherence rates to study procedures

Of the 20 participants enrolled in the THINK-MED RCT, 10 (50%) participants (*n* = 9 MCI participants; *n* = 1 SCI participants) withdrew from the study. From ‘THINK-MED Resource Staged’, 4 participants (*n* = 3 MCI, *n* = 1 SCI) withdrew from the study—1 before their baseline appointment (due to poor health), 1 due to health reasons and 2 as they were no longer interested in continuing with the study and reported the study visits to be too time consuming. Other reasons for withdrawal from the study included bereavement and not used to planning or cooking their own meals (*n* = 1 MCI, THINK-MED Resource Baseline), a diagnosis of AD during the study (*n* = 1 MCI control) and a further 4 no longer wished to continue as not interested in the research study (*n* = 4 MCI, *n* = 3 control, *n* = 1 THINK-MED Resource Baseline). This highlights the complexities associated with this older adult group in terms of the wider factors that may influence their ability to make dietary and lifestyle behaviour changes. Furthermore, in relation to those who withdrew from the control group as they were no longer interested, it is possible their decision was influenced by a lack of allocation to an intervention arm and this had diminished their willingness to continue. From review of the researcher field notes, *n* = 2 MCI participants (THINK-MED Resource Staged) indicated at their 6-month appointment that they were happy to continue with the study assessments; however, they were not willing to make any changes to their diet. By 12 months, these participants had withdrawn from the study. Thus, the most common reason for withdrawal from the study (*n* = 6/10 participants) was having no further interest in continuing with the dietary intervention study/making dietary changes.

#### Findings related to the suitability of data collection procedures and outcome measures

The outcome visits were conducted in the morning and required approximately 3–3.5 hours to complete all assessments. Overall, participants were able to complete the measures required in terms of their comprehension, understanding and capacity to undertake the measures. All MCI participants enrolled in the THINK-MED study had outcome assessments conducted in their own home, which was favoured by participants. Furthermore, as most of these MCI participants were retired, it allowed flexibility with appointments as they were readily available during the week. The feasibility of biological sample collection is an important consideration for the design of future trials, particularly among older adults. Scheduling/conducting the appointments in the participant’s home allowed the fasting blood sample to be taken at the beginning of the appointment, with adequate time for the participant to have a comfort break and have breakfast. At baseline, for some participants (*n* = 4), the researcher was unable to obtain blood sample due to difficulty finding veins. In comparison to the THINK-MED MCI study, arranging this appointment was more challenging for some SCI participants, as 2 participants were in full-time employment/full-time voluntary work. Therefore, appointments had to be scheduled at the weekend or earlier in the morning to facilitate work commitments. The feasibility of carrying out a 3.5-hour study appointment is an important factor to consider for future study planning. Indeed, the duration of study visits should be refined with a clear prioritisation of outcome measures required.

Time and response burden in relation to completing the 12 questionnaires for the THINK-MED study was heterogeneous between participants. Some participants required more assistance than others to complete the questionnaires, some needed encouragement and supervision while others benefited from having the questions read out loud. This ultimately increased the time required to complete the appointment. To facilitate this and reduce the time burden, the researcher agreed with the participant that any uncompleted questionnaires at the appointment could be returned to the researcher via post, with a stamped, addressed envelope provided. Some, but not all participants, who were offered this approach returned the necessary paperwork; however, 3 MCI participants (27%) failed to return at least one questionnaire and/or food diary. Furthermore, although the researcher tried to review all questionnaires for completeness, 5 MCI participants forgot to answer some questions or left some responses blank (on at least one questionnaire). This resulted in missing data (5.1%) across all of the outcome information collected.

Generally, the use of the CANTAB cognitive test battery was user-friendly and well-received. Some challenges were encountered as a result of completing the tests in the home environment, as some participants (*n* = 3) were distracted during some of the cognitive tasks (doorbell ringing, dog barking or telephone call) which may have affected their ability to perform at an optimal level in the tests. Indeed, all participants completed the seven cognitive tasks, with the exception of 1 MCI participant who did not complete one out of the seven tasks (DMS) as they were distracted and wished to proceed to the next task. Some participants commented that the automated instructions provided by the CANTAB app were not always clear or were relayed too fast in order for them to comprehend what was required. However, prior to each task the researcher provided an overview of the upcoming task to the participant, which was then followed by the automated instructions.

##### Study evaluation questionnaire

The participants were asked to complete an evaluation questionnaire at the end of the study. Within this questionnaire, participants were asked to provide feedback on data collection/outcome assessments, elements of the intervention and acceptability towards the dietary changes. Feedback was based on a 5-point Likert-type scale, with a score closer to 5.0 representing a more positive experience. All of the MCI participants who completed the 12-month follow-up, completed the evaluation questionnaire. Across all MCI participants, the median score (on the Likert-type scale) was 4.5, 5.0, 5.0 and 4.0, respectively, for the experience associated with providing blood samples, having blood pressure taken, having anthropometric measurement taken and completing questionnaires/outcome assessments. The MCI participants in the THINK-MED Resource and THINK-MED Resource Staged groups scored a median of 4.5, 3.5, 3.0 and 3.0 when asked about elements of intervention such as providing appropriate information at the beginning of the study, motivation to continue the study, the recipe booklets and the shopping list, respectively. Among the MCI participants in the THINK-MED Resource and THINK-MED Resource Staged groups, 50% of them stated that they would continue the MD lifestyle. The feasibility of adding fruits and vegetables, olive oil, oily fish, nuts and whole grains to their diet was also rated highly (median values were 5.0, 4.5, 4.0, 3.5 and 4.0, respectively).

All of the SCI participants (*n* = 4) who completed the 6-month follow-up, completed the evaluation questionnaire. Evaluations were rated highly in terms of the experience associated with providing blood samples, having blood pressure taken, having anthropometric measurement taken and completing questionnaires/outcome assessments (median scores were 4.0, 4.0, 4.5 and 4.0, respectively). Similarly, feedback from the SCI participants in the THINK-MED Resource and THINK-MED Resource Staged groups in relation to elements of the intervention, provision of appropriate information at the beginning of the study, motivation to continue the study, the recipe booklets and the shopping list were also highly rated with scores of 5.0, for each measure, respectively. Encouragingly, all of the SCI participants in the THINK-MED Resource and THINK-MED Resource Staged groups stated that they would continue the MD lifestyle. The feasibility of adding fruits and vegetables, olive oil, oily fish, nuts and whole grains to their diet was also rated highly with scores of 5.0 for each measure, respectively.

##### Mediterranean Diet Score (MDS)

MDS from baseline to 6 months and 12 months for the THINK-MED MCI participants is presented in Table 4. At baseline, MDSs were 3.0, 2.0 and 3.0 for THINK-MED Resource, THINK-MED Resource Staged and control groups, respectively. For those in the intervention groups, MDS increased by 6 months (8.0 for THINK-MED Resource vs 5.0 for THINK-MED Resource Staged) with a slight drop in score for the control group (2.0). By 12 months, those in the intervention groups appeared to maintain the change in MDS seen at 6 months. The control group did seem to increase in MDS by 12 months; however, this was only a median change of 1.5 points overall from baseline. MDS from baseline to 6 months for the THINK-MED SCI participants are also presented in Table 4. At baseline, median MDSs were 3.0, 3.0 and 6.0 for the THINK-MED Resource, THINK-MED Resource Staged and control groups, respectively. By 6 months (end of study), improvements in score were demonstrated by both intervention groups (10.0 THINK-MED Resource vs 10.0 THINK-MED Resource Staged). The control group showed a drop in median MDS from 6.0 to 4.0 by 6 months. It is important to note that the control group had a relatively high MDS at baseline, possibly due to the commencement of dietary changes post-screening. The drop in MDS by 6 months suggests that these changes were not sustained.

**Table 4:** Changes in Mediterranean Diet Scores (MDS) from baseline to 6 months and 12 months assessment visit for participants with MCI (*n* = 14, as *n* = 1 withdrew before baseline)

	MCI participants (***n*** = 14)			SCI participants (***n*** = 5)		
Participant Group	Group 1 (THINK-MED resource) (***n*** = 5)	Group 2 (THINK-MED resource staged) (***n*** = 3)	Group 3 (control) (***n*** = 6)	Group 1 (THINK-MED resource) (***n*** = 2)	Group 2 (THINK-MED resource staged) (***n*** = 2)	Group 3 (control) (***n*** = 1)
**MDS at baseline** (median (min, max))	3.0 (3.0,4.0)	2.0 (0.0,2.0)	3.0 (2.0,4.0)	3.0 (2.0,3.0)	3.0 (1.0,5.0)	6.0 (5.0,6.0)
**MDS at 6 months** (median (min, max))	8.0 (3.0,9.0)^a^	5.0 (4.0,5.0)	2.0 (2.0,5.0)^c^	10.0 (8.0,11.0)	10.0 (10.0,10.0)^b^	4.0 (4.0,4.0)
**MDS at 12 months** (median (min, max))	8.0 (3.0,9.0)^a^	5.0 (5.0,5.0)^b^	6.0 (5.0,6.0)^**d**^	–	–	–
**Change in MDS from baseline to 6 months** (median (min, max))	5.0^a^ (0.0,5.0)	3.0 (3.0,4.0)	0.0 (-2.0,1.0)^c^	7.0 (6.0,8.0)	5.0 (5.0,5.0)^b^	-4.0 (-4.0,-4.0)
**Change in MDS from 6 months to 12 months** (median (min, max))	0.0^a^ (-1.0,1.0)	1.0 (1.0,1.0)	2.0 (0.0,4.0)^**d**^	–	–	–
**Change in MDS from baseline to 12 months** (median (min, max))	4.0^a^ (0.0,6.0)	5.0 (5.0,5.0)^b^	1.5 (1.0,2.0)^**d**^	–	–	–

^a^*n* = 3, ^b^*n* = 1, ^c^*n* = 3, ^d^*n* = 2

Reference range 0–14, with higher scores indicating higher adherence to a MD

## Discussion

This current study provides a response to calls for the development and testing of preventative interventions to reduce the risk of cognitive decline [60]. We assessed the feasibility of recruitment into, and retention of, participants in the THINK-MED dietary intervention among a clinical sample of MCI patients and a community-based sample of participants with SCI. This research is important as it can inform future research and is relevant to researchers interested in enrolling participants at risk of cognitive decline into clinical trials. In summary, taking into account the challenges encountered with the recruitment of MCI patients, inability to meet the desired sample size and retention challenges faced during implementation of this pilot study, a tailored MD intervention among a MCI population may not be feasible. The qualitative study conducted in Phase I of this research provided indications that participants with MCI may be motivated to change their diet towards a MD and helped to shape the materials to support behaviour change. In Phase II, there were some indications of improvement in MDS during the intervention and some willingness to continue with MD dietary changes among those who completed the study. However, recruitment and retention, especially from a MCI clinical population, was extremely challenging, highlighting the individuality and complex needs of a clinical cohort such as those with MCI. Recruitment from a community-based sample with SCI may be more viable because this approach can reach a wider audience of individuals with less complex health needs, in a shorter time period. However, the subjective nature of this target group and absence of a confirmed, clinical diagnosis of cognitive impairment is a clear limitation of this approach. Similar recruitment challenges of MCI patients were experienced by a multi-center NeuroExercise trial in Germany, the Netherlands and Ireland, who also found recruitment from a memory clinic setting difficult and slow. However, offering the same intervention at a non-medical site was much more successful and a more attractive approach than recruitment through the memory clinics [61].

The feasibility experiences of the THINK-MED study have provided some valuable lessons in terms of study design and have revealed some areas that require improvement and refinement for future research. Recommendations, with discussion of supporting rationale, to refine the study design and procedures for further pilot and feasibility testing of this intervention could include:

- Review of the inclusion and exclusion criteria for entry into the study to ensure that the criteria used is appropriate and non-restrictive, particularly in relation to MD intake on entry, consumption of nutritional supplements and exclusion of those with a BMI ≥ 40 kg/m^2^

We found that the MDS inclusion criteria for the THINK-MED study may have been too stringent for recruitment of participants into the trial. However, it is important to consider that, if the MDS for inclusion had been higher, it may have allowed enrolment of participants who were already consuming the main elements of a MD, and therefore, they would not have had as much scope to change their diet towards a MD as someone who had a lower score on the scale. The PREDIMED trial [16, 17] is one of the largest RCTs to test the effectiveness of a MD with cognitive function outcomes and for this intervention study, benefits of adherence to a MD where shown among participants with a higher baseline MDS (approximately 8+ points) [62]. It should be noted however that this trial took place in a Mediterranean country where consumption of a MD is more common. A recently published single group, uncontrolled feasibility study (*n* = 26), ‘Brainfood’, focusing on promotion of a MD combined with physical activity and mindfulness strategies over a 5-week intervention period in people with MCI/mild dementia, enrolled participants with a mean MDS of 8.0 points at baseline, showing significant increases of 2.0 points post-intervention (*p* = 0.002) [63]. Moreover, in the UK, the MED-EX trial, an Alzheimer’s Research UK funded study, aims to test the feasibility (with a future definitive trial planned) of a MD intervention, combined with physical activity, among people at risk of cognitive decline [64]. The authors have indicated that, in regard to MD eligibility criteria, participants with a MDS of less than nine will be eligible—a MDS criteria five points greater than that tested here in the THINK-MED study. Going forward, for designing future MD trials, it would be important to consider the appropriate cutoffs for MDS to ensure the criteria is not too restrictive yet allows maximum benefit of the intervention to those enrolled.

A further decision regarding eligibility to participate relates to the consumption of nutritional supplements. Any benefits in cognitive function associated with a greater adherence to a MD could be potentially overshadowed by the beneficial effects of a nutritional supplement, such as Souvenaid. For the THINK-MED study, owing to the potential benefits in cognitive function of Souvenaid, as shown by the Lipidiet study [59] (cognitive function benefits shown by secondary outcomes only) and the associated cost involved in purchasing this supplement, the research team in consultation with memory clinic physicians, felt it unethical to ask participants to stop taking this supplement or be a reason to prevent them from taking part. This is an important consideration for future trials of this nature. We also found that when recruiting from the community, some participants were ineligible for inclusion as they scored below the MoCA cutoff score for SCI. As the target sample for this study was mainly older adults, this is something that may be unavoidable and not necessarily something that should be altered for future studies. It may however depend on the recruitment sampling frame used, as mentioned previously some older adult charities/services supported people who were more dependent and had poorer functionality; therefore, may have had poorer cognitive health. The use of a risk score may be worth incorporating into a larger evaluative trial. For example, in the FINGER study [65], the CAIDE (Cardiovascular Risk Factors, Aging and Incidence of Dementia) risk score was used, which is based on parameters for age, hypertension, hypercholesterolemia, physical inactivity, obesity and educational level [66]. Those who had a CAIDE score of ≥ 6 points, indicating the presence of modifiable risk factors and cognitive performance at the mean level or slightly lower than expected for age were included. Furthermore, in the proposed MED-EX UK feasibility study [64], the authors plan to recruit people with a CVD risk profile using the QRISK3 score, with eligibility for those with a score of 15 or above. Therefore, inclusion of a dementia risk scoring tool may be a more appropriate approach for screening and recruiting from this target sample.

Finally, some participants were excluded from the THINK-MED study due to their BMI. This criteria was used in response to challenges encountered with obtaining blood samples from those in higher BMI categories in previous dietary intervention studies [44]. This may be something that is very individual and not experienced by all so, and in terms of a main trial, this criterion could be adjusted in order to maximise recruitment.

b) Prioritisation of core outcome measures owing to the occurrence of missing data experienced with the outcome measures assessed in this pilot RCT may be required to maximise completeness and reduce time/response burden on participants, perhaps focussing on MD behaviour change and self-efficacy

As discussed, the data collection experiences encountered in the THINK-MED study led to the occurrence of missing data across some of the outcome assessments, making comparisons between time-points and intervention groups limited. For further feasibility and pilot testing, it may be imperative to streamline or prioritise the questionnaire data collected, perhaps including MDS and MD behaviour change focussing specifically on the barriers to changing towards a MD and self-efficacy. This would also reduce the time burden experienced by some participants, as the outcome assessment visits for the THINK-MED study were lengthy and may have acted as a barrier for continued participation in this study. By doing so, this may increase the retention of participants in a study of this nature. For future trials, ensuring the incorporation of a logic model from the conceptualisation of the study design is important in terms of determining feasibility of outcome measures, as they can help prioritise and structure data collection and analysis. Inclusion of pre-defined progression criteria would also be of value. These data can be used to explain how the intervention works to achieve its outcomes or help to understand if outcomes cannot be met [67].

c) Review of the target population and sampling frame used for recruitment owing to the difficulties encountered recruiting from a clinical population of MCI patients.

Recruitment of people at risk of cognitive decline from a clinical setting was challenging; however, using a community-based sample may be an alternative approach. It will require appropriate planning to ensure the provision of an adequate time-frame to recruit participants, identification of the availability of organisations and methods used to promote and advertise the research. Encouraging dietary behaviour change, and sustaining the changes made, is challenging in any population; however, the experiences encountered during the THINK-MED study highlight the heterogeneity of the MCI population, with many factors influencing their ability to agree to participate in and remain compliant during the study and make the required dietary changes. It was evident that as the study progressed some older individuals were not willing to make any dietary changes, as they were happy with their current routine. Similar findings were expressed in a qualitative study among British adults who indicated that changing to MD, in a non-Mediterranean country, could be difficult to adapt as they associated a MD with increased cost, increased food preparation time and difficulty in sourcing the required foods [68]. A report by Mody et al. (2008) [69] discussed similar challenges with recruitment and retention of older adults into clinical research studies. The study highlighted that, although it is imperative to research this population to inform clinical practice, this cohort are often under-represented in intervention studies owing to this group having increased likelihood of complex comorbidities, sometimes lower motivation to participate in research and some experiencing emotional burden [69]. Thus, the nature and complexity of this population group could suggest that participation in, and sustainability of achieving dietary and lifestyle behaviour change, is extremely individual and could be influenced by personal circumstances. In addition to the complex needs of this heterogeneous clinical cohort, there were also difficulties accessing and recruiting these patients through memory clinics as discussed previously. Going forward, it might be useful to have dedicated staff, like a clinical research nurse, who can review case notes and identify potentially eligible patients. This could offload the burden from clinicians.

Following recruitment for the THINK-MED SCI study, it was encouraging that a larger number of people were informed about the study in a shorter timeframe. That said, the numbers enrolled in the study were small. Using a group setting to promote the THINK-MED SCI study was beneficial in terms of reaching a larger number of people at once. The study researcher was able to provide an overview of the research to a small group of people on one occasion and then liaised individually with those who were interested in finding out further information. This was useful in order to establish a rapport with participants, and an effective way to judge participant suitability for the study rather than solely advertising the research by poster. The researcher had the opportunity to answer any questions that were raised by the group about the research, something that would have added extra time if participants were contacted individually. Furthermore, the researcher was able to seek advice from relevant staff within each organisation as to the suitability of certain individuals to participate for the study. On the other hand, in comparison to MCI recruitment from a clinical setting, it was necessary to include an additional screening tool to assess cognitive function for SCI participants. Data collectors must be fully trained in the use of such tools and factor adequate time to conduct these assessments. However, a cognitive assessment tool like the MoCA used here is a short, simple questionnaire and can be easily implemented. These considerations provide important learning points for further feasibility and pilot testing, reinforcing the need to ensure the sampling frame used for the study is appropriate and that other recruitment options outside of a clinical setting are explored and thoroughly planned.

### Strengths and limitations

To our knowledge, this study is among the first pilot MD interventions aimed at MCI participants and those at risk of cognitive decline in the UK, providing novel and important evidence in an area where more primary research is needed. The phased approach of intervention development, through to assessment of feasibility and acceptability by pilot study is in accordance with established guidelines [38, 39]. Participatory involvement of MCI patients, in the initial evaluation of MD educational material [41] and during the refinement phase (Phase I) of the work presented in this paper, adds robustness to the design and development of the intervention resources. The small sample size for this pilot study, together with the number of participants who withdrew from the study and level of missing data encountered during data collection, is a clear limitation to this research. Nonetheless, it provides important learning points for other researchers in this field who are interested in the recruitment of older adults with, or at risk of, cognitive impairment into clinical trials.

## Conclusion

With the growth of an aging population and the risk of cognitive decline increasing in line with age, encouraging behaviour change towards a MD and its associated lifestyle has the potential to improve cognition and overall health of our older generations. The research presented in this paper has contributed to the growing MD and ageing evidence base. A MD resource was developed and evaluated by participants with cognitive impairment. This provided important understandings in relation to the barriers and enablers to dietary and lifestyle change among older adults with MCI and will be of importance to researchers and clinicians working with this cohort. Pilot testing revealed that recruitment and retention in a dietary intervention, especially from a MCI clinical population, was extremely challenging and highlighted the complex needs of a clinical population such as those with MCI. Recruitment from a community-based sample with SCI may be more viable, as this approach can reach a wider audience of individuals in a shorter time period. This paper has provided a number of important considerations to inform the design of future dietary intervention studies in this field.

## Supplementary Information

**Additional file 1: Table S1.:** Theoretical Domains Framework applied to structured interview schedule (Michie et al., 2014). **Table S2.** Results for the THINK-MED participants with MCI according to various cognitive tasks (n=14, as n=1 withdrew before baseline). **Table S3.** Results for the THINK-MED participants with SCI according to various cognitive tasks (n=5). THINK-MED Resources and poster advertisement

## Data Availability

The datasets generated during and/or analysed during the current study are not publicly available but are available from the corresponding author on reasonable request.

## Abbreviations

MD: Mediterranean diet
MDS: Mediterranean Diet Score
MCI: Mild cognitive impairment
SCI: Subjective cognitive impairment
AD: Alzheimer’s disease
BMI: Body mass index
NI: Northern Ireland
