# Supplementary material for: A mixed methods pilot randomised controlled trial to develop and evaluate the feasibility of a Mediterranean diet and lifestyle education intervention ‘THINK-MED’ among people with cognitive impairment
Source: Pilot Feasibility Stud. 2021 Jan 4;7:3. doi: 10.1186/s40814-020-00738-3 (PMC7780397; doi:10.1186/s40814-020-00738-3)
Supplement: Supplementary file 1 — Additional file 1: Table S1.: Theoretical Domains Framework applied to structured interview schedule (Michie et al., 2014). Table S2. Results for the THINK-MED participants with MCI according to various cognitive tasks (n=14, as n=1 withdrew before baseline). Table S3. Results for the THINK-MED participants with SCI according to various cognitive tasks (n=5). THINK-MED Resources and poster advertisement [file 40814_2020_738_MOESM1_ESM.docx]

| **Supplementary Material Table 1** : Theoretical Domains Framework applied to structured interview schedule (Michie et al., 2014) | | |
| --- | --- | --- |
| **TDF Domain** | **Definition and Theoretical Constructs represented within each domain (adapted from Michie et al., 2014, pp. 88-90)** | **Example question from Structured Interview** |
| Knowledge | An awareness of something (including knowledge of condition/scientific rationale) | Before you received the resource, had you heard about the Mediterranean diet and the types of foods it recommends? Do you think there is a link between diet and lifestyle and memory? |
| Skills | An ability or proficiency acquired through practice (including skills, skills development and competence) | Can you tell us how the Mediterranean diet differs from what you currently eat?  What is your opinion about the recipe books? How might these be useful? (Who does most of the shopping and cooking in then household?) |
| Goals | Mental representation of outcomes or end states that an individual wants to achieve (including goal/target setting and action planning) | The resource recommends setting small personal dietary goals towards a Mediterranean diet. How do you feel this approach might help people change their diet? |
| Memory, attention and decision processes | The ability to retain information, focus selectively on aspects of the environment and choose between two or more alternatives (including memory, attention, cognitive overload) | What did you think of the format and layout of the resource?  Would the calendar and memory book be something you might use? Do you think this resource would be useful for people with mild memory problems? |
| Behavioural regulation | Anything aimed at managing or changing objectively observed or measured actions (including self-monitoring, breaking habit and action planning) | The resource recommends setting small personal dietary goals towards a Mediterranean diet? How do you feel this approach might help people change their diet? |
| Environmental context and resources | Any circumstance of a person’s situation or environment that discourages or encourages the development of skills and abilities, independence, social competence and adaptive behaviour (including resources, material resources, barriers and facilitators) | Can you think of anything that would help to provide extra support to help you change your diet and lifestyle?  Is there anything you didn’t like about the resource? What would you change about it? |
| Intention | A conscious decision to perform a behaviour or resolve an act in a certain way (stages of change model and transtheorectical model) | Would you like to change to a more Mediterranean way of eating- if not, why not? Have you made any changes since reading the resource? (What types of changes? Or would you plan to make any changes in the future?) |
| Optimism | The confidence that things will happen for the best or that the desired goals will be attained | After reading the resource, do you think the resource would help people with memory problems change their diet? In what way? |
| Beliefs about the consequences | Acceptance of the truth, reality or validity about outcomes of a behaviour (including beliefs, outcome expectancies) | In your opinion, are there any consequences of not making changes to your diet and lifestyle? |
| Beliefs about capabilities | Acceptance of the truth, reality or validity about an ability, talent or facility that a person can put to constructive use (including self-confidence, self-efficacy, beliefs, empowerment) | In your opinion, what sorts of things could make it difficult for people with memory problems to change towards a Mediterranean diet? |
| Emotion | A complex reaction pattern, involving experiential, behavioural and physiological elements, by which the individual attempts to deal with a personally significant matter or event | Would you like to change to a more Mediterranean way of eating- if not, why not?  In your opinion, what sorts of things could make it difficult for people with memory problems to change towards a Mediterranean diet? |
| Reinforcement | Increasing the probability of a response by arranging a dependent relationship, or contingency, between the response and a given stimulus | Can you think of anything that would help to provide extra support to help you change your diet and lifestyle?  Would the calendar and memory book be something you might use? Do you think this resource would be useful for people with mild memory problems |

**Supplementary Material Table 2**: Results for the THINK-MED participants with MCI according to various cognitive tasks (n=14, as n=1 withdrew before baseline)

| **Measure of CANTAB Test** | **All THINK-MED (n=14)** | | | **Group 1: THINK-MED Resource at Baseline (n=5)** | | | **Group 2: THINK-MED Resourced Stage (n=3)** | | | **Group 3: Usual Care (n=6)** | | |
| --- | --- | --- | --- | --- | --- | --- | --- | --- | --- | --- | --- | --- |
|  | **Baseline**  **(n=14)** | **6 months**  **(n=9)** | **12 months**  **(n=6)** | **Baseline**  **(n=5)** | **6 months**  **(n=3)** | **12 months**  **(n=3)** | **Baseline**  **(n=3)** | **6 months**  **(n=3)** | **12 months**  **(n=1)** | **Baseline**  **(n=6)** | **6 months**  **(n=3)** | **12 months**  **(n=2)** |
| **Delayed Matching to Sample (DMS)** |  |  |  |  |  |  |  |  |  |  |  |  |
| *DMS - all trials (% correct)* |  |  |  |  |  |  |  |  |  |  |  |  |
| Mean | 67.9 | 67.5 | 65.0 | 68.8 | 71.7 | 71.7 | 63.3 | 62.5 |  | 70.0 | 66.7 | 55.0 |
| SD | 15.1 | 14.4 | 19.4 | 11.1 | 7.6 | 18.9 | 24.7 | 31.8 |  | 14.6 | 10.4 | 21.2 |
| Minimum | 35.0 | 40.0 | 40.0 | 55.0 | 65.0 | 50.0 | 35.0 | 40.0 |  | 50.0 | 55.0v | 40.0 |
| Maximum | 90.0 | 85.0 | 85.0 | 80.0 | 80.0 | 85.0 | 80.0 | 85.0 |  | 90.0 | 75.0 | 70.0 |
| *DMS - 0 (% correct) (0 Second delay)* |  |  |  |  |  |  |  |  |  |  |  |  |
| Mean | 58.3 | 70.0 | 65.0 | 60.0 | 73.3 | 61.7 | 46.7 | 60.0 |  | 64.0 | 73.3 | 70.0 |
| SD | 27.6 | 23.9 | 41.5 | 16.3 | 11.6 | 50.1 | 50.3 | 56.6 |  | 21.9 | 11.6 | 42.4 |
| Minimum | 0.0 | 20.0 | 5.0 | 40.0 | 60.0 | 5.0 | 0.0 | 20.0 |  | 40.0 | 60.0 | 40.0 |
| Maximum | 100.0 | 100.0 | 100.0 | 80.0 | 80.0 | 100.0 | 100.0 | 100.0 |  | 100.0 | 80.0 | 100.0 |
| *DMS - 4 (% correct) (4 Second delay)* |  |  |  |  |  |  |  |  |  |  |  |  |
| Mean | 66.7 | 57.5 | 48.2 | 65.0 | 60.0 | 53.7 | 60.0 | 80.0 |  | 72.0 | 40.0 | 40.0 |
| SD | 19.7 | 24.9 | 33.1 | 25.2 | 20.0 | 45.6 | 20.0 | 28.3 |  | 17.9 | 20.0 | 0.0 |
| Minimum | 40.0 | 20.0 | 1.0 | 40.0 | 40.0 | 1.0 | 40.0 | 20.0 |  | 60.0 | 20.0 | 0.0 |
| Maximum | 100.0 | 100.0 | 80.0 | 100.0 | 80.0 | 80.0 | 80.0 | 60.0 |  | 100.0 | 60.0 | 0.0 |
| *DMS - 12 (% correct) (12 Second delay)* |  |  |  |  |  |  |  |  |  |  |  |  |
| Mean | 63.3 | 55.0 | 32.0 | 60.0 | 60.0 | 40.0 | 73.3 | 40.0 |  | 60.0 | 60.0 | 20.0 |
| SD | 16.7 | 20.7 | 33.5 | 16.3 | 20.0 | 40.0 | 23.1 | 28.3 |  | 14.1 | 20.0 | 28.3 |
| Minimum | 40.0 | 20.0 | 1.0 | 40.0 | 40.0 | 0.0 | 60.0 | 20.0 |  | 40.0 | 40.0 | 0.0 |
| Maximum | 100.0 | 80.0 | 80.0 | 80.0 | 80.0 | 80.0 | 100.0 | 60.0 |  | 80.0 | 80.0 | 40.0 |
| *DMS - all delays (% correct) (0,4,12 second)* |  |  |  |  |  |  |  |  |  |  |  |  |
| Mean | 62.7 | 60.9 | 49.2 | 61.5 | 61.5 | 64.3 | 60.0 | 60.0 |  | 65.2 | 58.0 | 43.5 |
| SD | 15.0 | 17.8 | 31.6 | 10.1 | 10.1 | 10.3 | 24.3 | 38.2 |  | 14.7 | 15.6 | 23.3 |
| Minimum | 33.0 | 33.0 | 6.0 | 53.0 | 53.0 | 53.0 | 33.0 | 33.0 |  | 53.0 | 40.0 | 27.0 |
| Maximum | 87.0 | 87.0 | 87.0 | 73.0 | 73.0 | 73.0 | 80.0 | 87.0 |  | 87.0 | 80.0 | 60.0 |
| **Motor Screening (MOT)** |  |  |  |  |  |  |  |  |  |  |  |  |
| *MOT Mean Latency (ms)* |  |  |  |  |  |  |  |  |  |  |  |  |
| Mean | 1033.0 | 994.6 | 1008.5 | 1140.8 | 964.4 | 873.5 | 935.8 | 1050.6 | 1605.2 | 991.9 | 968.7 | 912.6 |
| SD | 214.6 | 303.6 | 323.3 | 145.9 | 266.2 | 213.0 | 271.1 | 511.7 |  | 232.3 | 170.6 | 53.3 |
| Minimum | 709.1 | 662.2 | 665.4 | 963.1 | 662.2 | 665.4 | 773.4 | 702.6 | 1605.2 | 709.1 | 772.7 | 874.9 |
| Maximum | 1305.4 | 1638.1 | 1605.2 | 1305.4 | 1164.0 | 1164.0 | 1248.8 | 1638.1 | 1605.2 | 1303.4 | 1083.5 | 950.2 |
| **Paired Associates Learning (PAL)** |  |  |  |  |  |  |  |  |  |  |  |  |
| *PAL - First Attempt Memory Score (number of times per attempt)* |  |  |  |  |  |  |  |  |  |  |  |  |
| Median | 3.5 | 3.0 | 3.0 | 2.0 | 3.0 | 2.0 | 1.0 | 1.0 | 2.0 | 6.5 | 3.0 | 4.5 |
| Minimum | 0.0 | 0.0 | 0.0 | 2.0 | 2.0 | 0.0 | 0.0 | 0.0 | 2.0 | 1.0 | 0.0 | 4.0 |
| Maximum | 11.0 | 11.0 | 17.0 | 9.0 | 11.0 | 17.0 | 10.0 | 8.0 | 2.0 | 11.0 | 3.0 | 5.0 |
| *PAL - Total Errors (number of times incorrect choice)* |  |  |  |  |  |  |  |  |  |  |  |  |
| Median | 49.5 | 58.0 | 50.5 | 49.0 | 50.0 | 60.0 | 64.0 | 66.0 | 44.0 | 49.0 | 58.0 | 48.5 |
| Minimum | 17.0 | 12.0 | 3.0 | 28.0 | 12.0 | 3.0 | 18.0 | 12.0 | 44.0 | 17.0 | 52.0 | 40.0 |
| Maximum | 65.0 | 68.0 | 65.0 | 61.0 | 61.0 | 65.0 | 65.0 | 68.0 | 44.0 | 64.0 | 60.0 | 57.0 |
| **Pattern Recognition Memory (PRM)** |  |  |  |  |  |  |  |  |  |  |  |  |
| *PRM - Immediate (% correct)* |  |  |  |  |  |  |  |  |  |  |  |  |
| Mean | 81.4 | 69.4 | 63.9 | 77.1 | 72.2 | 66.7 | 88.9 | 69.4 | 83.3 | 80.56 | 66.7 | 50.0 |
| SD | 16.0 | 13.8 | 14.6 | 15.8 | 12.7 | 8.3 | 19.2 | 17.4 | 0.0 | 16.7 | 16.7 | 11.8 |
| Minimum | 58.3 | 50.0 | 41.7 | 66.7 | 58.3 | 58.3 | 66.7 | 50.0 | 83.3 | 58.3 | 50.0 | 41.7 |
| Maximum | 100.0 | 83.3 | 83.3 | 100.0 | 83.3 | 75.0 | 100.0 | 83.3 | 83.3 | 100.0 | 83.3 | 58.3 |
| *PRM - Delayed (% correct)* |  |  |  |  |  |  |  |  |  |  |  |  |
| Mean | 65.3 | 65.7 | 66.7 | 77.1 | 83.3 | 69.4 | 52.8 | 50.0 |  | 63.3 | 63.9 | 62.5 |
| SD | 15.8 | 22.6 | 10.2 | 8.0 | 22.1 | 12.7 | 12.7 | 25.0 |  | 17.3 | 9.6 | 5.9 |
| Minimum | 41.7 | 25.0 | 58.3 | 66.7 | 58.3 | 58.3 | 41.7 | 25.0 |  | 41.7 | 58.3 | 58.3 |
| Maximum | 83.3 | 100.0 | 83.3 | 83.3 | 100.0 | 83.3 | 66.7 | 75.0 |  | 83.3 | 75.0 | 66.7 |
| **Reaction Time (RTI)** |  |  |  |  |  |  |  |  |  |  |  |  |
| *RTI - Reaction Time (ms)* |  |  |  |  |  |  |  |  |  |  |  |  |
| Mean | 420.5 | 507.6 | 609.9 | 426.7 | 574.3 | 574.8 | 435.6 | 573.7 | 1014.6 | 408.7 | 374.7 | 460.2 |
| SD | 47.2 | 232.2 | 294.1 | 63.6 | 302.1 | 320.5 | 55.0 | 288.8 | 0.0 | 37.1 | 36.3 | 121.8 |
| Minimum | 356.2 | 332.9 | 374.0 | 372.0 | 399.1 | 374.3 | 382.4 | 405.8 | 1014.6 | 356.2 | 332.9 | 374.1 |
| Maximum | 514.9 | 923.2 | 1014.6 | 514.9 | 923.1 | 944.4 | 492.3 | 907.3 | 1014.6 | 492.3 | 396.8 | 546.3 |
| *RTI - Movement Time (ms)* |  |  |  |  |  |  |  |  |  |  |  |  |
| Mean | 327.9 | 344.6 | 448.4 | 296.1 | 377.3 | 419.7 | 325.5 | 366.8 | 741.7 | 350.5 | 289.7 | 344.8 |
| SD | 67.6 | 131.5 | 166.9 | 54.7 | 143.8 | 121.1 | 101.5 | 187.8 | 0.0 | 60.2 | 79.6 | 5.2 |
| Minimum | 246.0 | 202.1 | 320.9 | 246.0 | 256.9 | 320.9 | 260.9 | 255.7 | 741.7 | 249.7 | 202.1 | 341.2 |
| Maximum | 442.5 | 583.7 | 741.7 | 373.0 | 536.5 | 554.8 | 442.5 | 583.7 | 741.7 | 416.8 | 357.4 | 348.5 |
| **Rapid Visual Processing (RVP)** |  |  |  |  |  |  |  |  |  |  |  |  |
| *RVP - Mean Response Latency (ms)* |  |  |  |  |  |  |  |  |  |  |  |  |
| Mean | 647.4 | 713.0 | 596.9 | 634.6 | 726.2 | 568.7 | 730.8 | 800.4 |  | 614.0 | 612.5 | 639.1 |
| SD | 142.7 | 196.7 | 87.4 | 146.8 | 206.8 | 103.2 | 233.4 | 291.2 |  | 92.2 | 19.5 | 57.6 |
| Minimum | 468.0 | 474.6 | 485.3 | 468.0 | 548.3 | 485.3 | 479.0 | 474.6 |  | 481.2 | 598.3 | 598.4 |
| Maximum | 939.8 | 1035.4 | 684.1 | 815.7 | 953.1 | 684.1 | 939.8 | 1035.4 |  | 757.8 | 634.7 | 679.8 |
| **Spatial Working Memory (SWM)** |  |  |  |  |  |  |  |  |  |  |  |  |
| *SWM - Number of Errors* |  |  |  |  |  |  |  |  |  |  |  |  |
| Median | 24.0 | 23.0 | 23.0 | 21.0 | 23.0 | 24.0 | 19.0 | 26.0 | 13.0 | 26.0 | 20.0 | 27.0 |
| Minimum | 3.0 | 2.0 | 13.0 | 18.0 | 2.0 | 18.0 | 3.0 | 24.0 | 13.0 | 22.0 | 19.0 | 22.0 |
| Maximum | 32.0 | 31.0 | 32.0 | 32.0 | 23.0 | 25.0 | 31.0 | 31.0 | 13.0 | 28.0 | 23.0 | 32.0 |
| *SWM - Strategy Use* |  |  |  |  |  |  |  |  |  |  |  |  |
| Median | 10.0 | 11.0 | 10.0 | 10.0 | 10.0 | 10.0 | 11.0 | 11.0 | 4.0 | 9.5 | 11.0 | 10.5 |
| Minimum | 3.0 | 8.0 | 4.0 | 10.0 | 8.0 | 9.0 | 3.0 | 10.0 | 4.0 | 8.0 | 9.0 | 10.0 |
| Maximum | 13.0 | 12.0 | 12.0 | 11.0 | 11.0 | 12.0 | 11.0 | 12.0 | 4.0 | 13.0 | 11.0 | 11.0 |

DMS – Reference range: 0-100, with higher scores indicating better performances

MOT – Reference range: 0-6000, with lower scores indicating better performances

PAL – Reference ranges: PAL first attempt – 0-20, with higher scores indicating better performances; PAL total errors – 0-70, with lower scores indicating better performances

PRM – Reference range: 0-100, with higher scores indicating better performances

RTI – Reference ranges: RTI reaction time/ movement time – 100-5100 ms, with lower scores indicating better performances

RVP – Reference ranges: RVP mean response latency – 100-1900 ms, with lower scores indicating better performances

SWM – Reference ranges: SWM number of errors – 0-153, with lower scores indicating better performances; SWM Strategy use – 2-14, with lower scores indicating better performances

Grey shaded cells indicate missing data

The CANTAB results of the THINK-MED MCI study participants are shown in Table. Overall, it is difficult to reach a conclusion based on the study results for each of the study groups. The participants in the ‘THINK-MED’ Resource at Baseline’ showed a decline in Motor Screening Mean Latency and Rapid Visual Processing Mean Response Latency

**Table 3 :** Results for the THINK-MED participants with SCI according to various cognitive tasks (n=5)

| **Measure of CANTAB Test** | **All THINK-MED (n=5)** | | **Group 1: THINK-MED Resource at Baseline (n=2)** | | **Group 2: THINK-MED Resourced Stage (n=2)** | | **Group 3: Usual Care (n=1)** | |
| --- | --- | --- | --- | --- | --- | --- | --- | --- |
|  | **Baseline**  **(n=5)** | **6 months**  **(n=4)** | **Baseline**  **(n=2)** | **6 months**  **(n=2)** | **Baseline**  **(n=2)** | **6 months**  **(n=1)** | **Baseline**  **(n=1)** | **6 months**  **(n=1)** |
| **Delayed Matching to Sample (DMS)** |  |  |  |  |  |  |  |  |
| *DMS - all trials (% correct)* |  |  |  |  |  |  |  |  |
| Mean | 81.0 | 81.3 | 85.0 | 85.0 | 77.5 | 80.0 | 80.0 | 75.0 |
| SD | 4.2 | 4.9 | 0.0 | 7.1 | 3.5 | 0.0 | 0.0 | 0.0 |
| Minimum | 75.0 | 75.0 | 85.0 | 80.0 | 75 | 80.0 | 80.0 | 75.0 |
| Maximum | 85.0 | 90.0 | 85.0 | 90.0 | 80 | 80.0 | 80.0 | 75.0 |
| *DMS - 0 (% correct) (0 Second delay)* |  |  |  |  |  |  |  |  |
| Mean | 88.0 | 80.0 | 100.0 | 80.0 | 70 | 60.0 | 100.0 | 100.0 |
| SD | 17.9 | 23.1 | 0.0 | 28.3 | 14.1 | 0.0 | 0.0 | 0.0 |
| Minimum | 60.0 | 60.0 | 100.0 | 60.0 | 60 | 60.0 | 100.0 | 100.0 |
| Maximum | 100.0 | 100.0 | 100.0 | 100.0 | 80 | 60.0 | 100.0 | 100.0 |
| *DMS - 4 (% correct) (4 Second delay)* |  |  |  |  |  |  |  |  |
| Mean | 76.0 | 75.0 | 80.0 | 70.0 | 70 | 100.0 | 80.0 | 60.0 |
| SD | 8.9 | 19.2 | 0.0 | 14.1 | 14.1 | 0.0 | 0.0 | 0.0 |
| Minimum | 60.0 | 60.0 | 80.0 | 60.0 | 60 | 100.0 | 80.0 | 60.0 |
| Maximum | 80.0 | 100.0 | 80.0 | 80.0 | 80 | 100.0 | 80.0 | 60.0 |
| *DMS - 12 (% correct) (12 Second delay)* |  |  |  |  |  |  |  |  |
| Mean | 60.0 | 75.0 | 60.0 | 90.0 | 70 | 60.0 | 40.0 | 60.0 |
| SD | 14.1 | 19.2 | 0.0 | 14.1 | 14.1 | 0.0 | 0.0 | 0.0 |
| Minimum | 40.0 | 60.0 | 60.0 | 80.0 | 60 | 60.0 | 40.0 | 60.0 |
| Maximum | 60.0 | 100.0 | 60.0 | 100.0 | 80 | 60.0 | 40.0 | 60.0 |
| *DMS - all delays (% correct) (0,4,12 second)* |  |  |  |  |  |  |  |  |
| Mean | 74.6 | 75.0 | 80.0 | 80.0 | 70 | 73.0 | 73.0 | 73.0 |
| SD | 8.9 | 19.5 | 0.0 | 9.9 | 4.2 | 0.0 | 0.0 | 0.0 |
| Minimum | 60.0 | 60.0 | 80.0 | 73.0 | 67 | 73.0 | 73.0 | 73.0 |
| Maximum | 80.0 | 100.0 | 80.0 | 87.0 | 73 | 73.0 | 73.0 | 73.0 |
| **Motor Screening (MOT)** |  |  |  |  |  |  |  |  |
| *MOT Mean Latency (ms)* |  |  |  |  |  |  |  |  |
| Mean | 895.7 | 873.5 | 954.0 | 938.4 | 891.4 | 832.0 | 787.6 | 785.4 |
| SD | 160.5 | 98.2 | 180.4 | 105.0 | 228 | 0.0 | 0.0 | 0.0 |
| Minimum | 730.2 | 785.4 | 826.4 | 864.1 | 730.2 | 832.0 | 787.6 | 785.4 |
| Maximum | 1081.5 | 1012.6 | 1081.5 | 1012.6 | 1052.6 | 832.0 | 787.6 | 785.4 |
| **Paired Associates Learning (PAL)** |  |  |  |  |  |  |  |  |
| *PAL - First Attempt Memory Score (number of times per attempt)* |  |  |  |  |  |  |  |  |
| Median | 9.0 | 8.0 | 16.0 | 8.0 | 4.5 | 10.0 | 9.0 | 6.0 |
| Minimum | 2.0 | 6.0 | 14.0 | 7.0 | 2 | 10.0 | 9.0 | 6.0 |
| Maximum | 18.0 | 10.0 | 18.0 | 9.0 | 7 | 10.0 | 9.0 | 6.0 |
| *PAL - Total Errors (number of times incorrect choice)* |  |  |  |  |  |  |  |  |
| Median | 29.0 | 40.0 | 15.0 | 31.0 | 50 | 41.0 | 29.0 | 47.0 |
| Minimum | 6.0 | 23.0 | 6.0 | 23.0 | 42 | 10.0 | 9.0 | 6.0 |
| Maximum | 58.0 | 47.0 | 24.0 | 39.0 | 58 | 10.0 | 9.0 | 6.0 |
| **Pattern Recognition Memory (PRM)** |  |  |  |  |  |  |  |  |
| *PRM - Immediate (% correct)* |  |  |  |  |  |  |  |  |
| Mean | 90.0 | 91.7 | 91.7 | 95.8 | 87.5 | 100.0 | 91.7 | 75.0 |
| SD | 7.0 | 11.8 | 11.8 | 5.9 | 5.9 | 0.0 | 0.0 | 0.0 |
| Minimum | 83.3 | 75.0 | 83.3 | 91.7 | 83.3 | 100.0 | 91.7 | 75.0 |
| Maximum | 100.0 | 100.0 | 100.0 | 100.0 | 91.7 | 100.0 | 91.7 | 75.0 |
| *PRM - Delayed (% correct)* |  |  |  |  |  |  |  |  |
| Mean | 68.3 | 89.6 | 75.0 | 87.5 | 70.8 | 100.0 | 50.0 | 83.3 |
| SD | 22.7 | 12.5 | 35.4 | 17.7 | 17.7 | 0.0 | 0.0 | 0.0 |
| Minimum | 50.0 | 75.0 | 50.0 | 75.0 | 58.3 | 100.0 | 50.0 | 83.3 |
| Maximum | 100.0 | 100.0 | 100.0 | 100.0 | 83.3 | 100.0 | 50.0 | 83.3 |
| **Reaction Time (RTI)** |  |  |  |  |  |  |  |  |
| *RTI - Reaction Time (ms)* |  |  |  |  |  |  |  |  |
| Mean | 407.6 | 414.0 | 447.2 | 451.1 | 388.7 | 342.0 | 366.5 | 410.5 |
| SD | 51.5 | 78.4 | 67.5 | 104.63 | 22.4 | 0.0 | 0.0 | 0.0 |
| Minimum | 366.5 | 342.0 | 399.4 | 377.1 | 372.8 | 342.0 | 366.5 | 410.5 |
| Maximum | 494.9 | 525.1 | 494.9 | 525.1 | 404.5 | 342.0 | 366.5 | 410.5 |
| *RTI - Movement Time (ms)* |  |  |  |  |  |  |  |  |
| Mean | 299.2 | 333.7 | 387.7 | 410.9 | 212.2 | 181.1 | 296.1 | 331.7 |
| SD | 139.9 | 146.6 | 217.4 | 171.2 | 12.6 | 0.0 | 0.0 | 0.0 |
| Minimum | 203.3 | 181.1 | 234.0 | 289.9 | 203.3 | 181.1 | 296.1 | 331.7 |
| Maximum | 541.4 | 531.9 | 541.4 | 531.9 | 221.1 | 181.1 | 296.1 | 331.7 |
| **Rapid Visual Processing (RVP)** |  |  |  |  |  |  |  |  |
| *RVP - Mean Response Latency (ms)* |  |  |  |  |  |  |  |  |
| Mean | 566.6 | 522.0 | 582.1 | 514.2 | 520.9 | 513.8 | 627.2 | 545.9 |
| SD | 73.3 | 16.0 | 103.6 | 3.0 | 49.1 | 0.0 | 0.0 | 0.0 |
| Minimum | 486.1 | 512.1 | 508.8 | 512.1 | 486.1 | 513.8 | 627.2 | 545.9 |
| Maximum | 655.4 | 545.9 | 655.4 | 516.3 | 555.6 | 513.8 | 627.2 | 545.9 |
| **Spatial Working Memory (SWM)** |  |  |  |  |  |  |  |  |
| *SWM - Number of Errors* |  |  |  |  |  |  |  |  |
| Median | 20.0 | 17.5 | 20.5 | 17.5 | 10.5 | 5.0 | 22.0 | 27.0 |
| Minimum | 8.0 | 5.0 | 20.0 | 15.0 | 8 | 5.0 | 22.0 | 27.0 |
| Maximum | 22.0 | 27.0 | 21.0 | 20.0 | 13 | 5.0 | 22.0 | 27.0 |
| *SWM - Strategy Use* |  |  |  |  |  |  |  |  |
| Median | 10.0 | 9.0 | 10.5 | 8.5 | 7.5 | 8.0 | 12.0 | 11.0 |
| Minimum | 6.0 | 7.0 | 10.0 | 7.0 | 6 | 8.0 | 12.0 | 11.0 |
| Maximum | 12.0 | 11.0 | 11.0 | 10.0 | 9 | 8.0 | 12.0 | 11.0 |

DMS – Reference range: 0-100, with higher scores indicating better performances

MOT – Reference range: 0-6000, with lower scores indicating better performances

PAL – Reference ranges: PAL first attempt – 0-20, with higher scores indicating better performances; PAL total errors – 0-70, with lower scores indicating better performances

PRM – Reference range: 0-100, with higher scores indicating better performances

RTI – Reference ranges: RTI reaction time/ movement time – 100-5100 ms, with lower scores indicating better performances

RVP – Reference ranges: RVP mean response latency – 100-1900 ms, with lower scores indicating better performances

SWM – Reference ranges: SWM number of errors – 0-153, with lower scores indicating better performances; SWM Strategy use – 2-14, with lower scores indicating better performances

**Supplementary material:** THINK-MED Resources

| 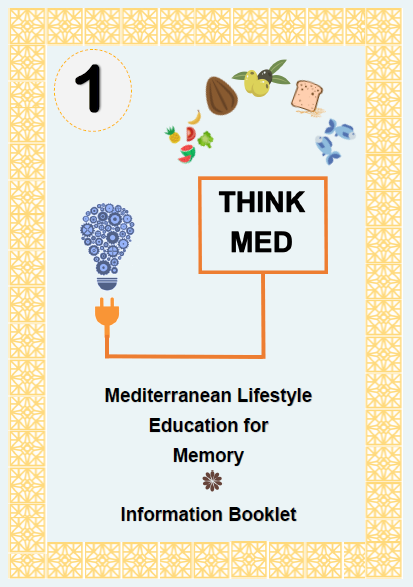 | 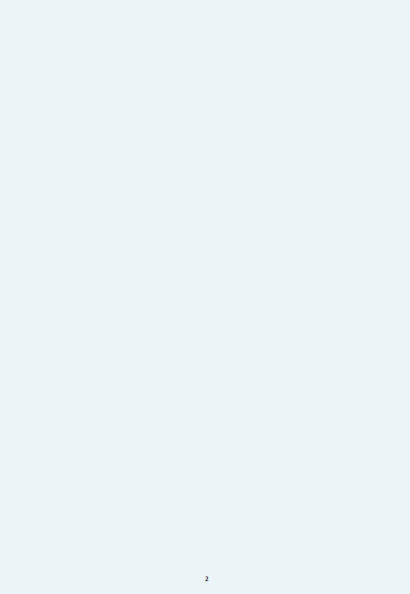 |
| --- | --- |
| 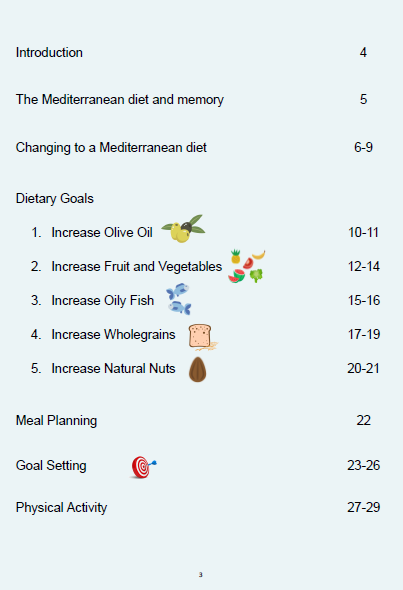 | 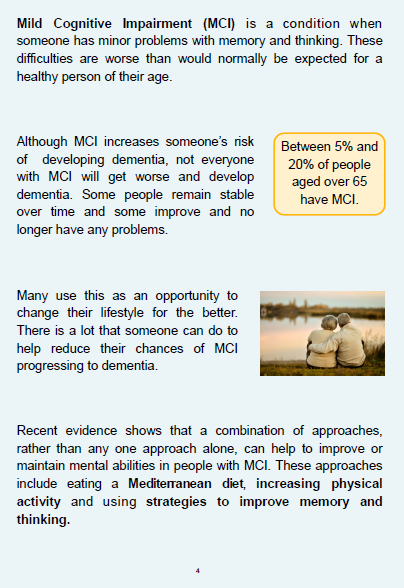 |
| 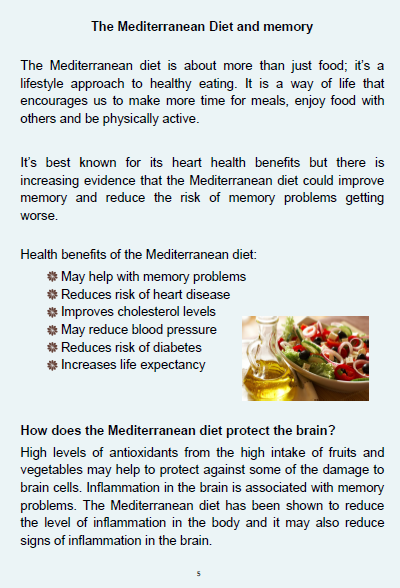 | 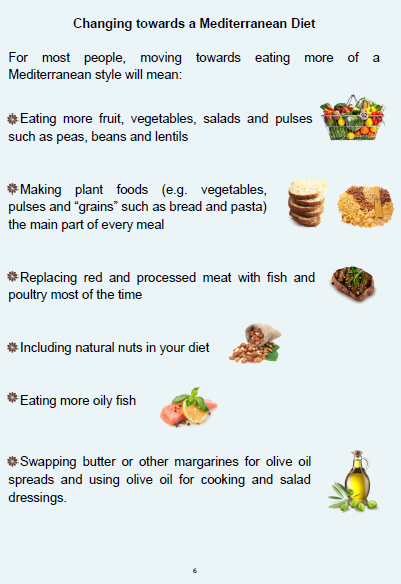 |
| 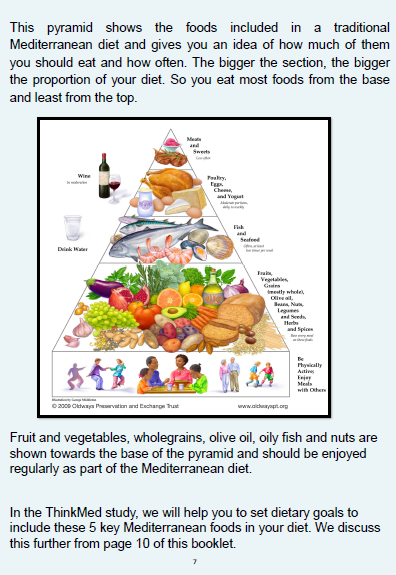 | 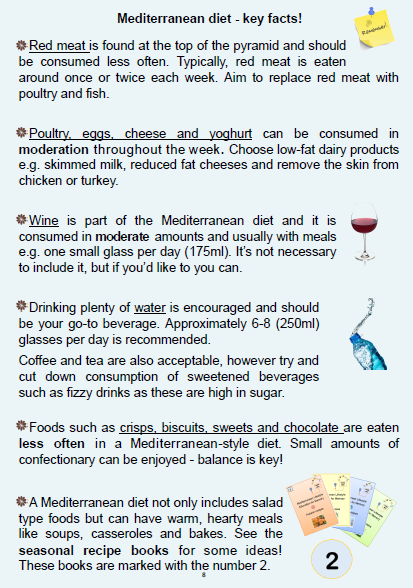 |
| 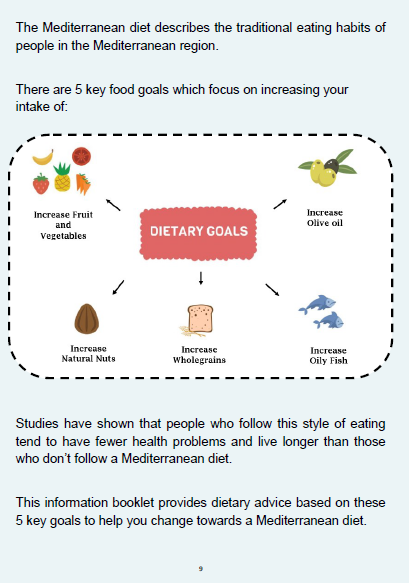 | 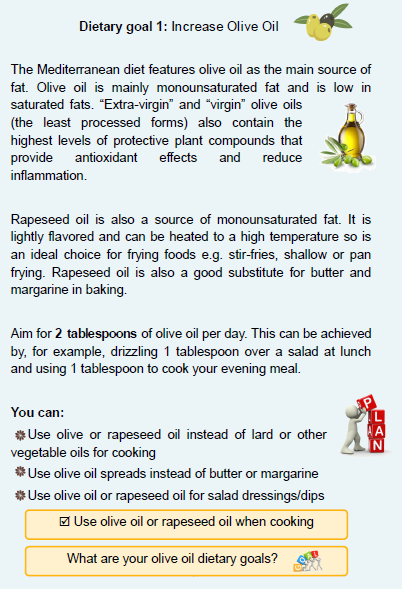 |
| 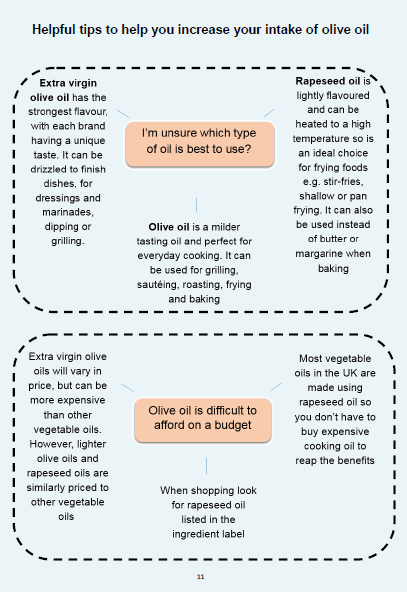 | 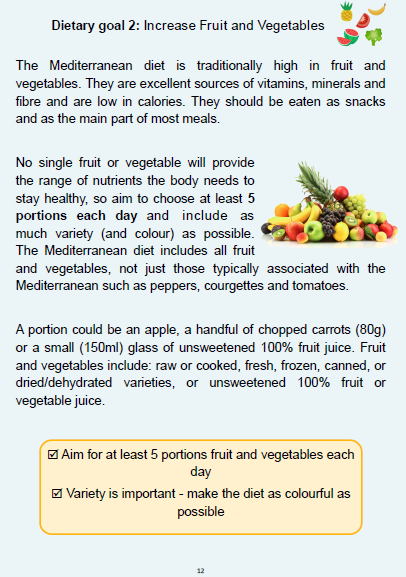 |
| 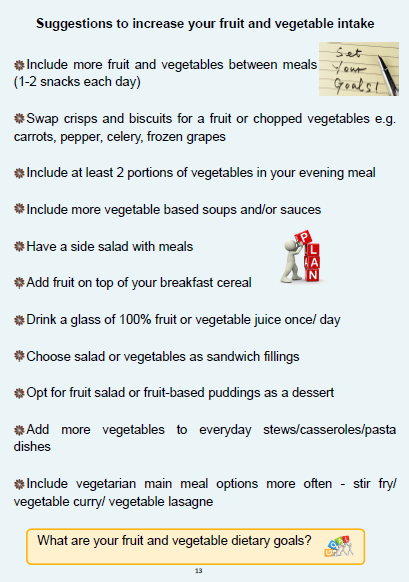 | 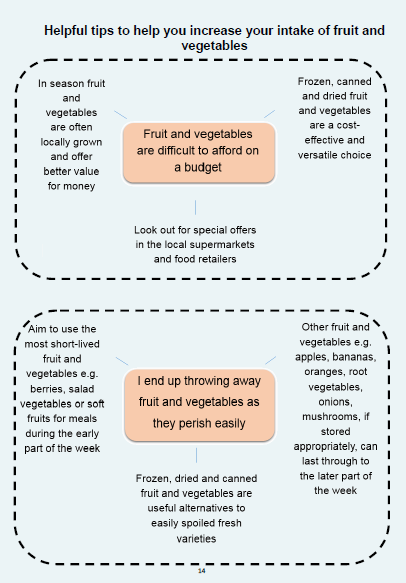 |
| 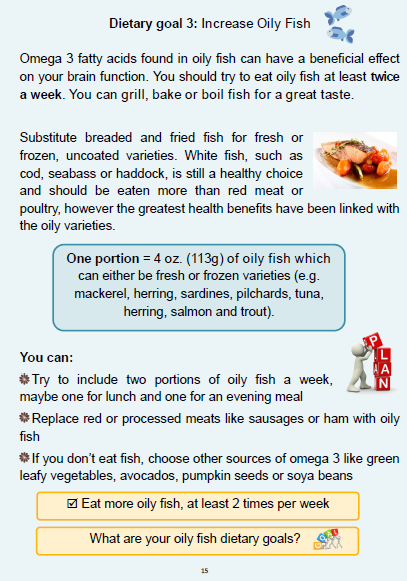 | 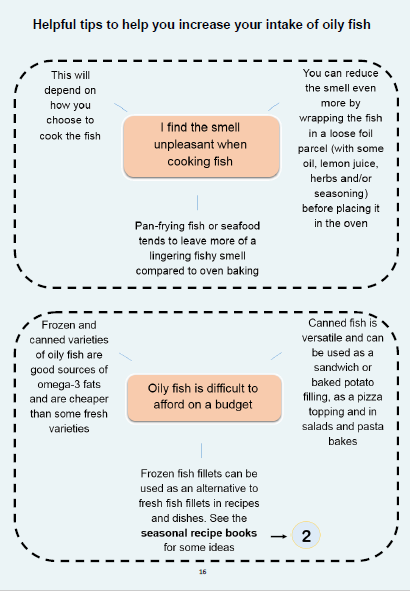 |
| 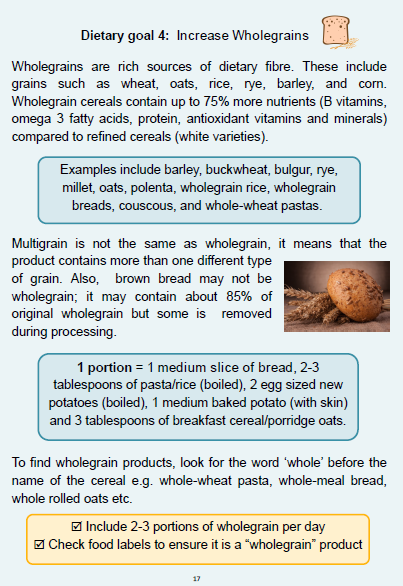 | 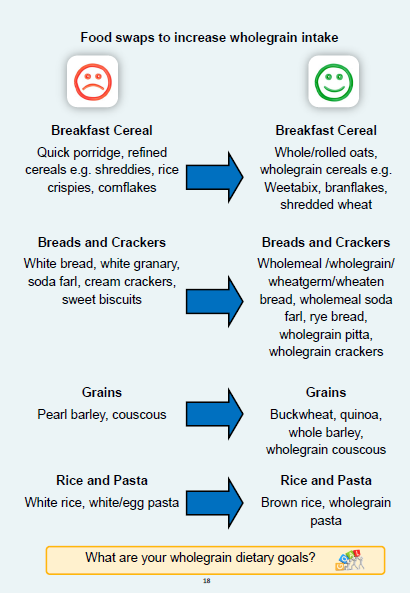 |
| 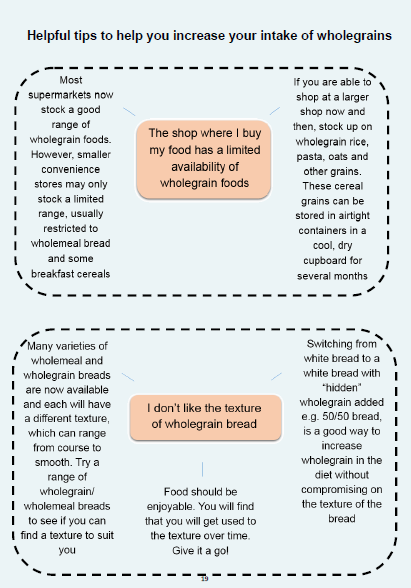 | 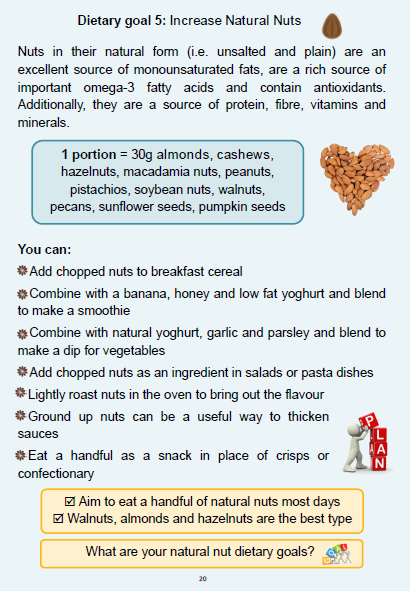 |
| 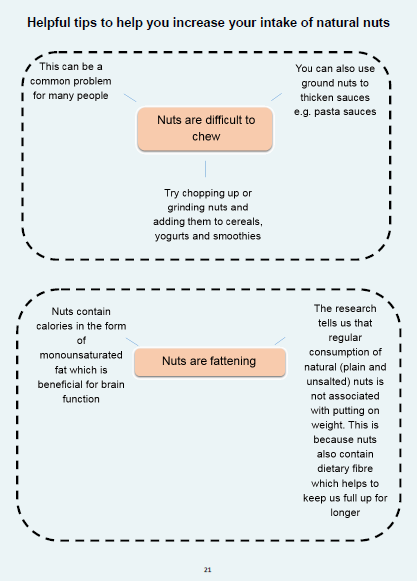 | 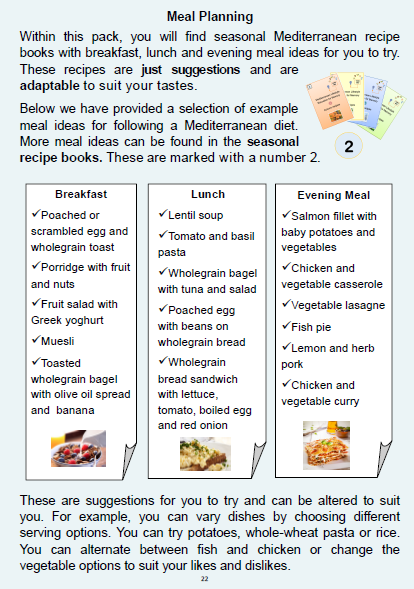 |
| 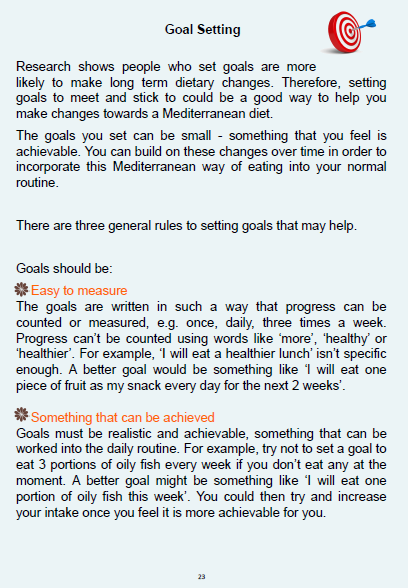 | 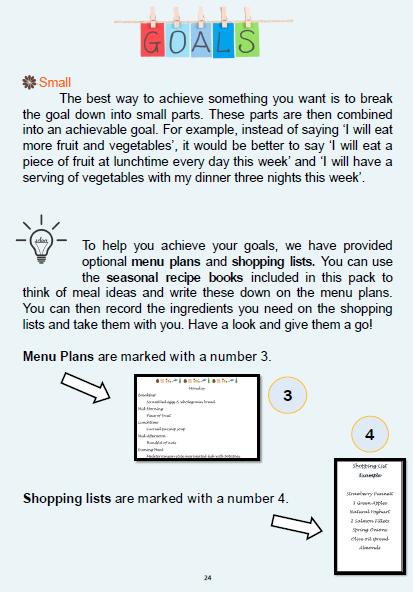 |
| 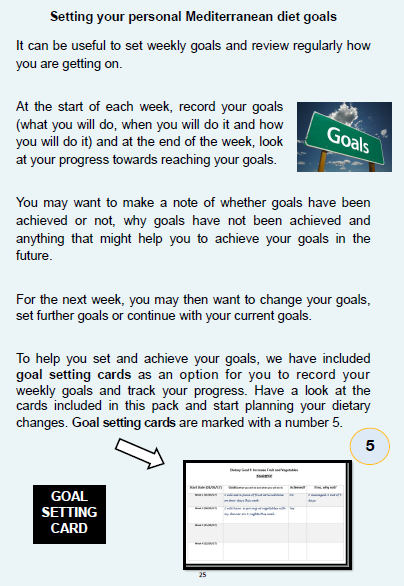 | 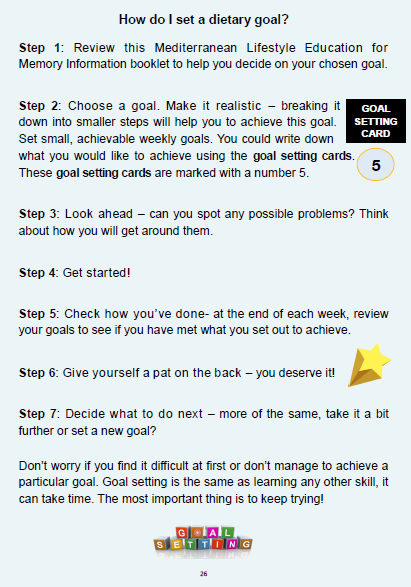 |
| 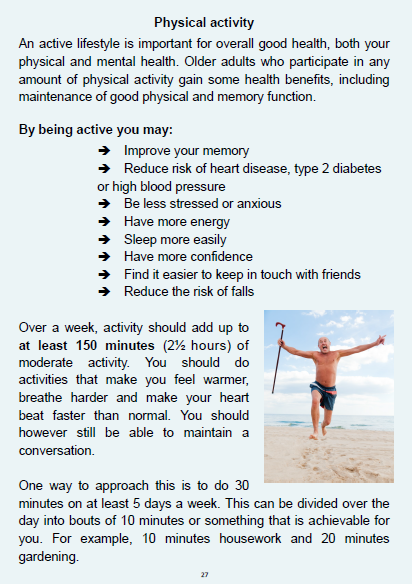 | 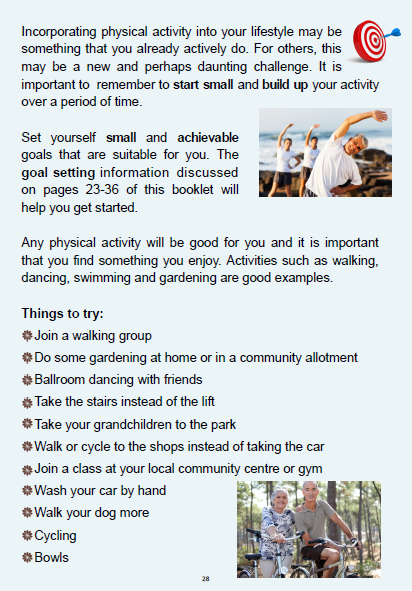 |
| 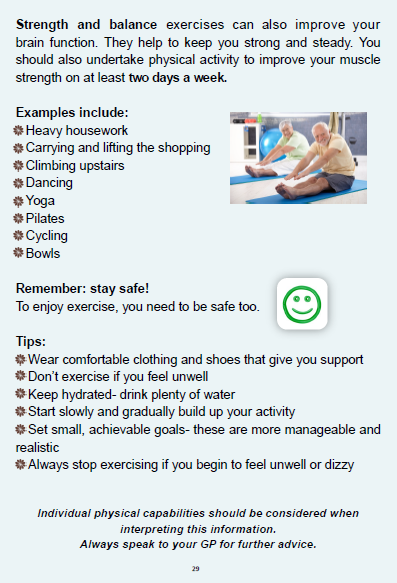 | 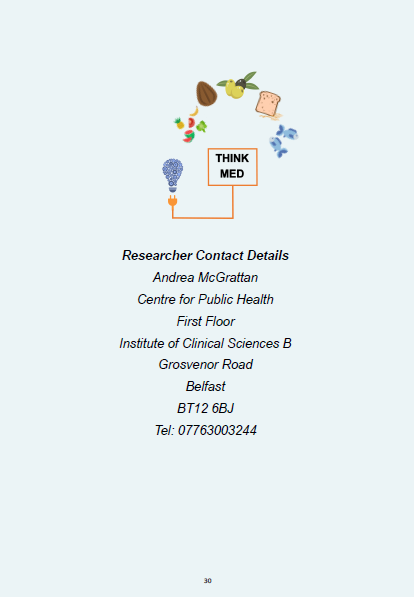 |
| 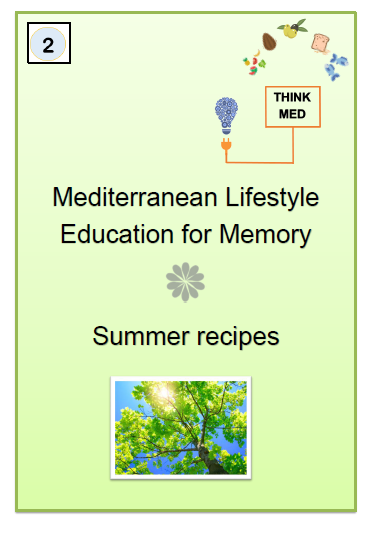 | 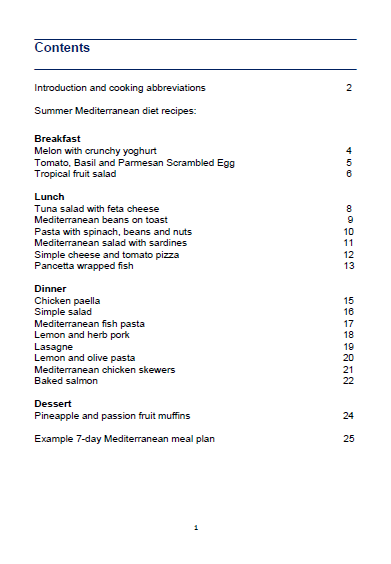 |
| 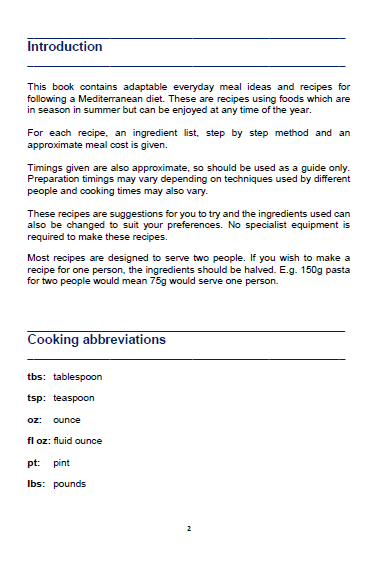 | 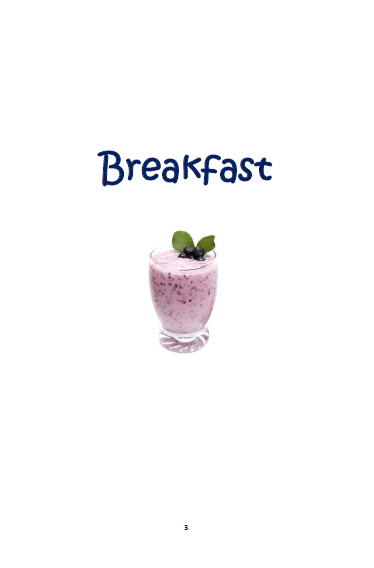 |
| 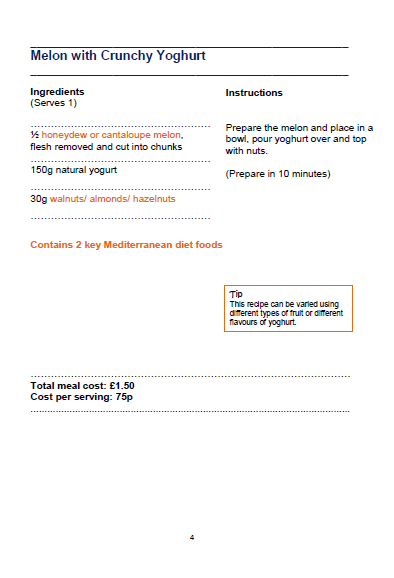 | 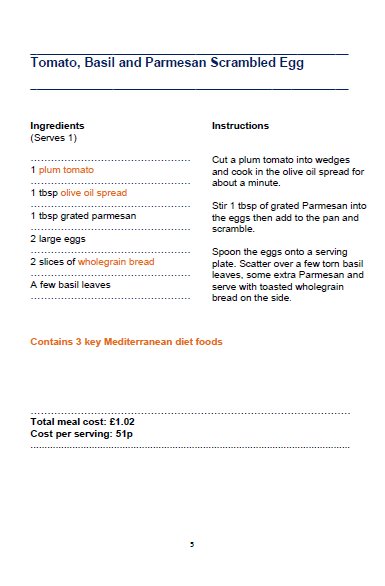 |
| 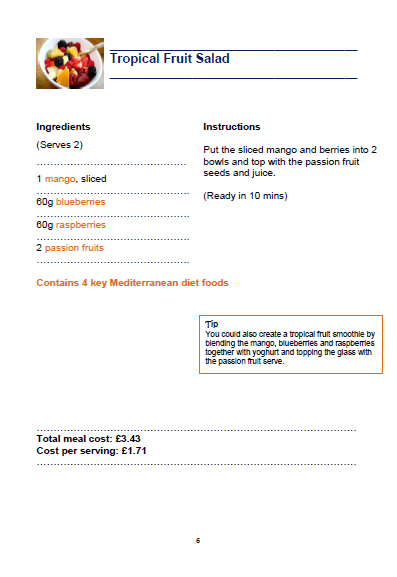 | 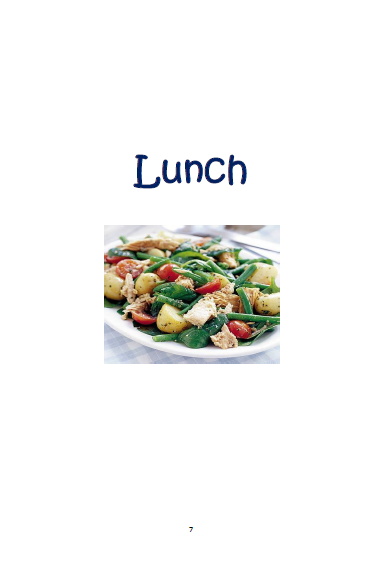 |
| 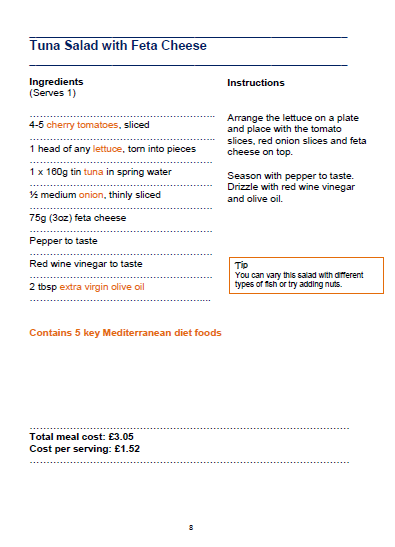 | 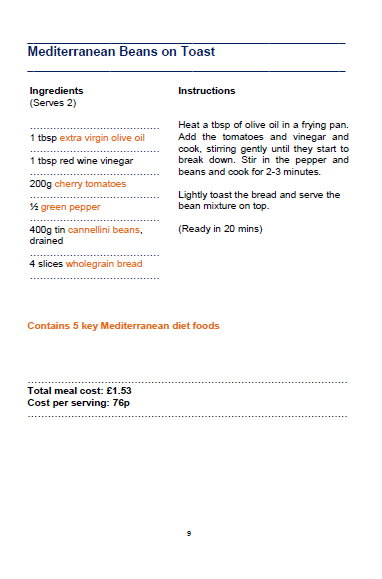 |
| 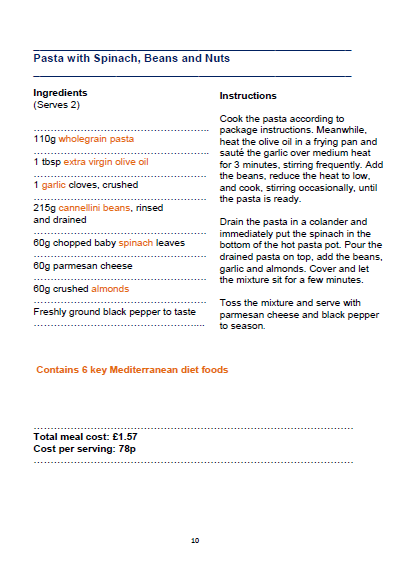 | 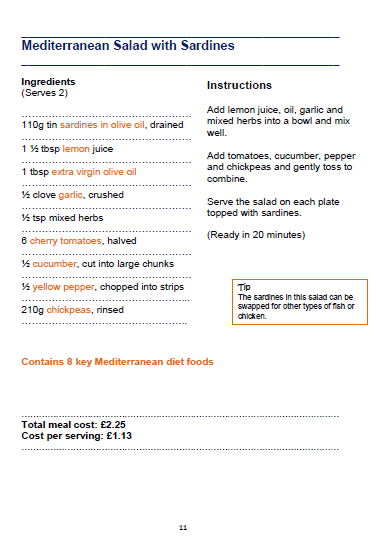 |
| 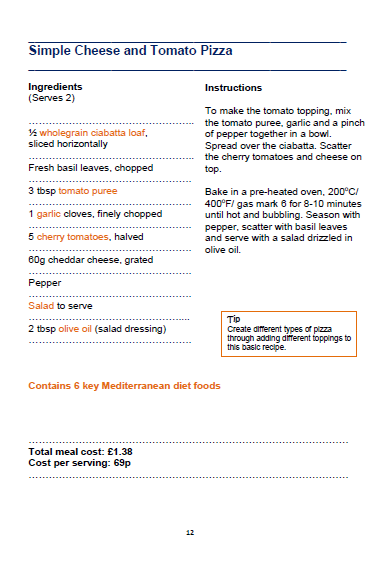 | 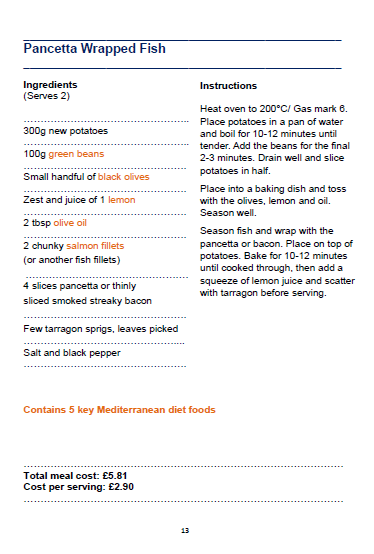 |
| 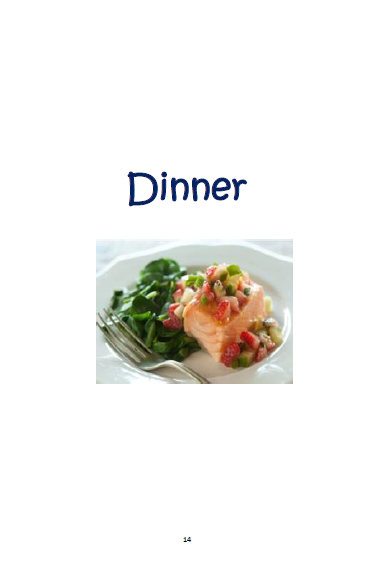 | 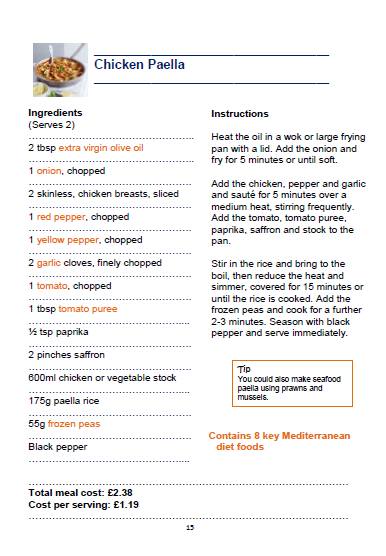 |
| 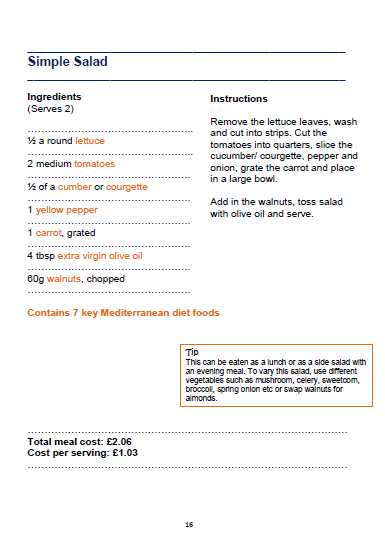 | 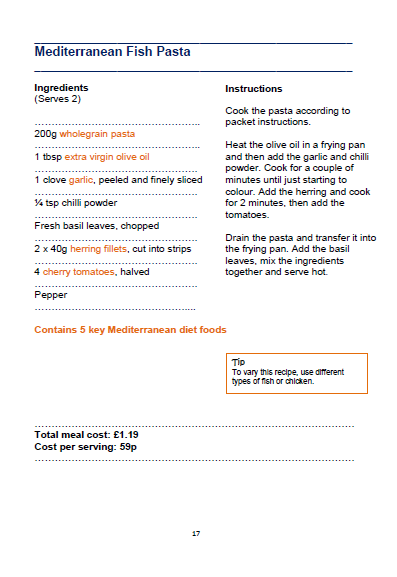 |
| 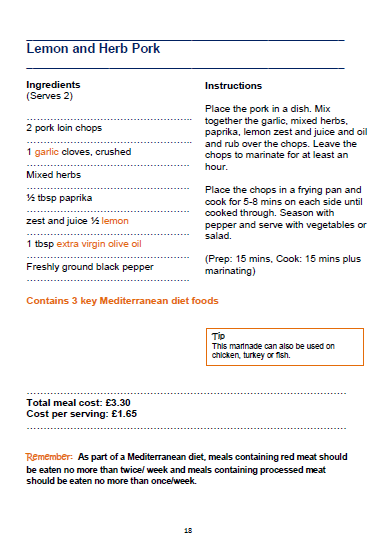 | 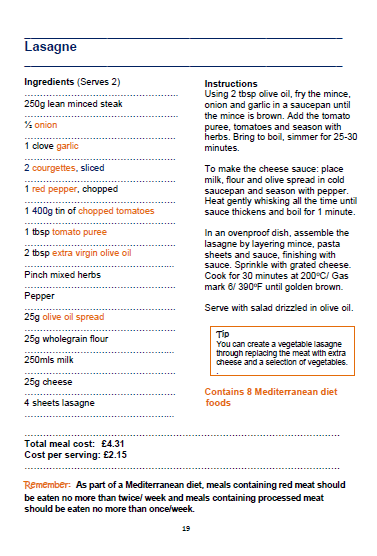 |
| 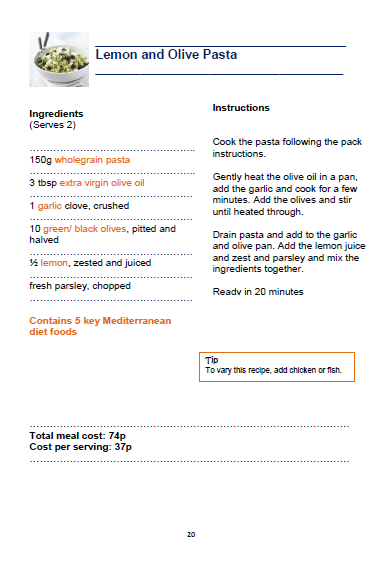 | 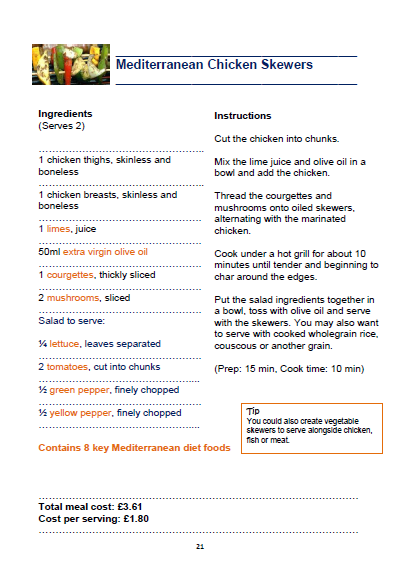 |
| 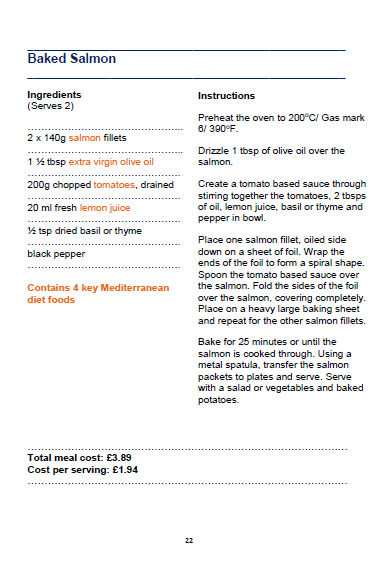 | 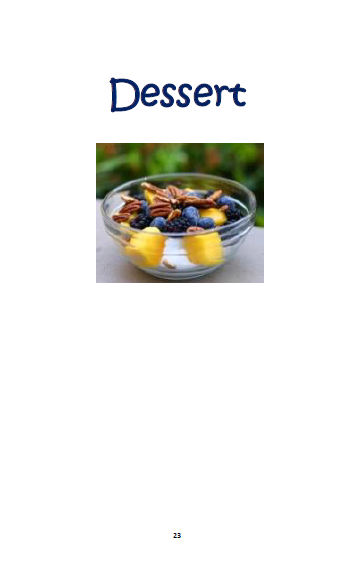 |
| 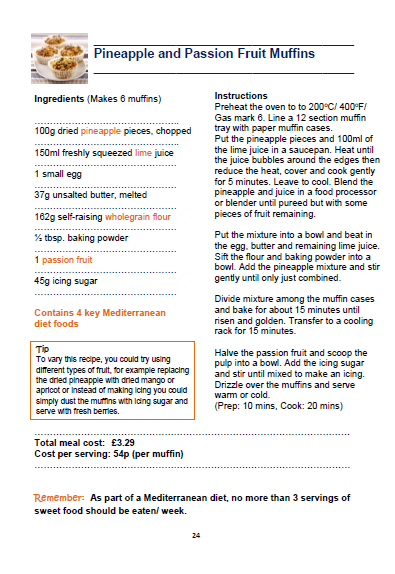 | 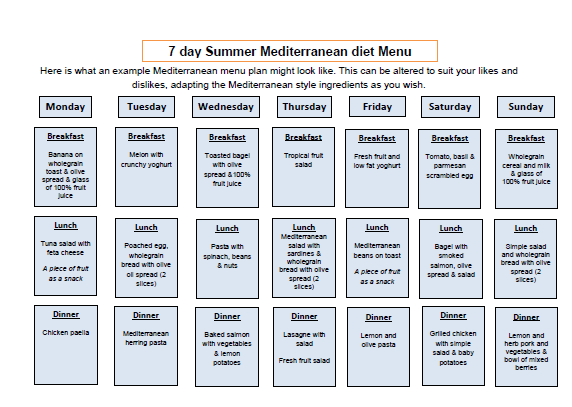 |
| 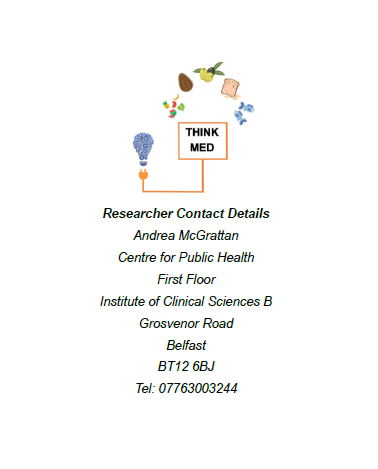 | 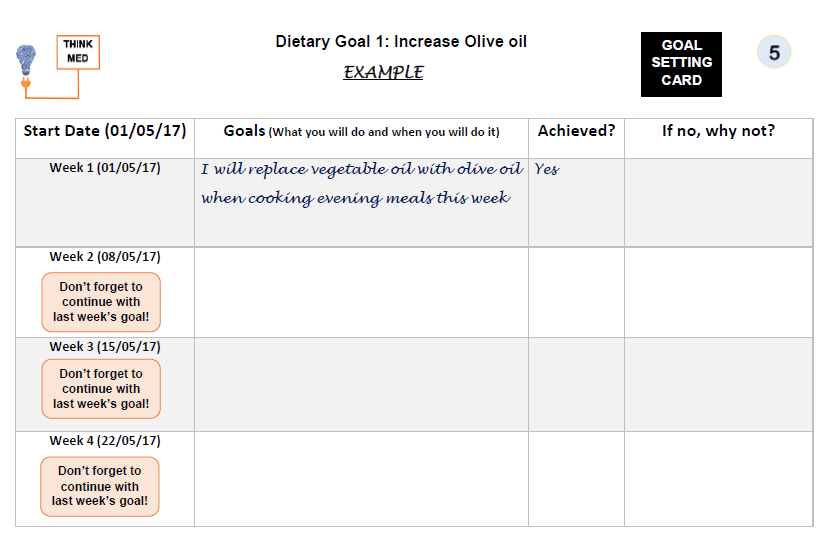 |
| 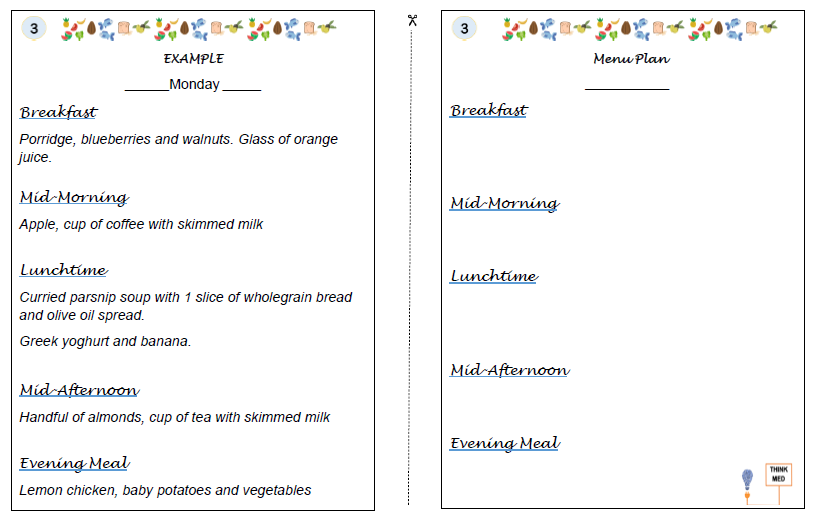 | 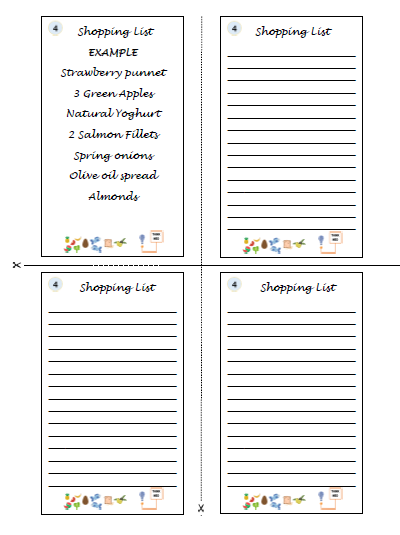 |

**Supplementary Material**: SCI Poster Advertisement


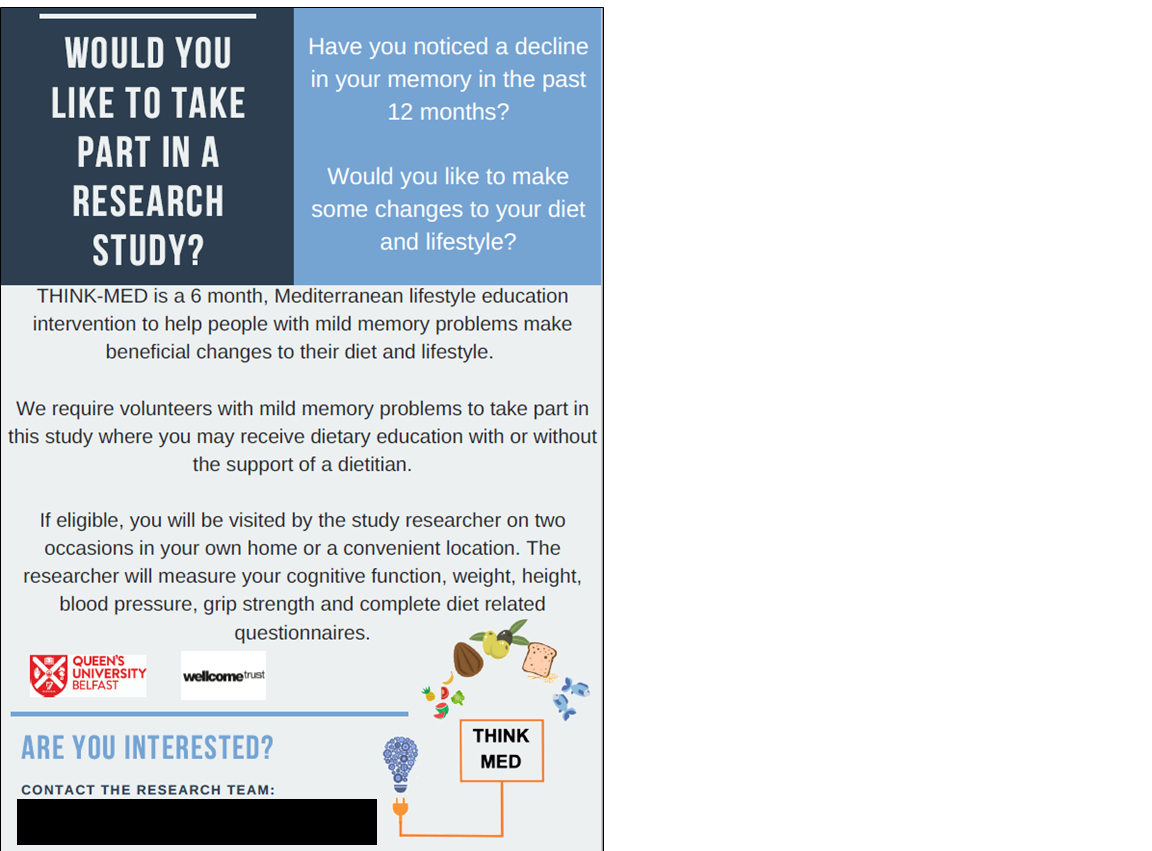


*Researcher contact details have been omitted
